# The Effect of Souvenaid on Functional Brain Network Organisation in Patients with Mild Alzheimer’s Disease: A Randomised Controlled Study

**DOI:** 10.1371/journal.pone.0086558

**Published:** 2014-01-27

**Authors:** Hanneke de Waal, Cornelis J. Stam, Marieke M. Lansbergen, Rico L. Wieggers, Patrick J. G. H. Kamphuis, Philip Scheltens, Fernando Maestú, Elisabeth C. W. van Straaten

**Affiliations:** 1 Alzheimer Center & Department of Neurology, Neuroscience Campus Amsterdam, VU University Medical Center, Amsterdam, The Netherlands; 2 Department of Clinical Neurophysiology, Neuroscience Campus Amsterdam, VU University Medical Center, Amsterdam, The Netherlands; 3 Nutricia Research, Utrecht, The Netherlands; 4 Laboratory of Cognitive and Computational Neuroscience, UCM-UPM Center for Biomedical Technology, Madrid, Spain; Cuban Neuroscience Center, Cuba

## Abstract

**Background:**

Synaptic loss is a major hallmark of Alzheimer’s disease (AD). Disturbed organisation of large-scale functional brain networks in AD might reflect synaptic loss and disrupted neuronal communication. The medical food Souvenaid, containing the specific nutrient combination Fortasyn Connect, is designed to enhance synapse formation and function and has been shown to improve memory performance in patients with mild AD in two randomised controlled trials.

**Objective:**

To explore the effect of Souvenaid compared to control product on brain activity-based networks, as a derivative of underlying synaptic function, in patients with mild AD.

**Design:**

A 24-week randomised, controlled, double-blind, parallel-group, multi-country study.

**Participants:**

179 drug-naïve mild AD patients who participated in the Souvenir II study.

**Intervention:**

Patients were randomised 1∶1 to receive Souvenaid or an iso-caloric control product once daily for 24 weeks.

**Outcome:**

In a secondary analysis of the Souvenir II study, electroencephalography (EEG) brain networks were constructed and graph theory was used to quantify complex brain structure. Local brain network connectivity (normalised clustering coefficient gamma) and global network integration (normalised characteristic path length lambda) were compared between study groups, and related to memory performance.

**Results:**

The network measures in the beta band were significantly different between groups: they decreased in the control group, but remained relatively unchanged in the active group. No consistent relationship was found between these network measures and memory performance.

**Conclusions:**

The current results suggest that Souvenaid preserves the organisation of brain networks in patients with mild AD within 24 weeks, hypothetically counteracting the progressive network disruption over time in AD. The results strengthen the hypothesis that Souvenaid affects synaptic integrity and function. Secondly, we conclude that advanced EEG analysis, using the mathematical framework of graph theory, is useful and feasible for assessing the effects of interventions.

**Trial registration:**

Dutch Trial Register NTR1975.

## Introduction

Worldwide, 35.6 million people suffer from dementia, of which Alzheimer’s disease (AD) is the most common form [1]. One of the earliest pathophysiological findings in AD is a loss of synapses [2], associated with degeneration of the synaptic membranes [3]. Synaptic loss may lead to impaired coupling between neurons, disrupting neuronal communication [4]. Episodic memory impairment is one of the earliest cognitive disturbances in the AD process [5], [6], and is associated with reduced number of synaptic contacts [7] and disturbed neuronal network communication [8]. Reducing synapse loss and improving synapse function may preserve or even improve neuronal communication and thereby positively affect memory and other cognitive functions. Loss of synapses and synapse function provide potentially useful targets for intervention in AD [9], [10].

**Figure 1:**
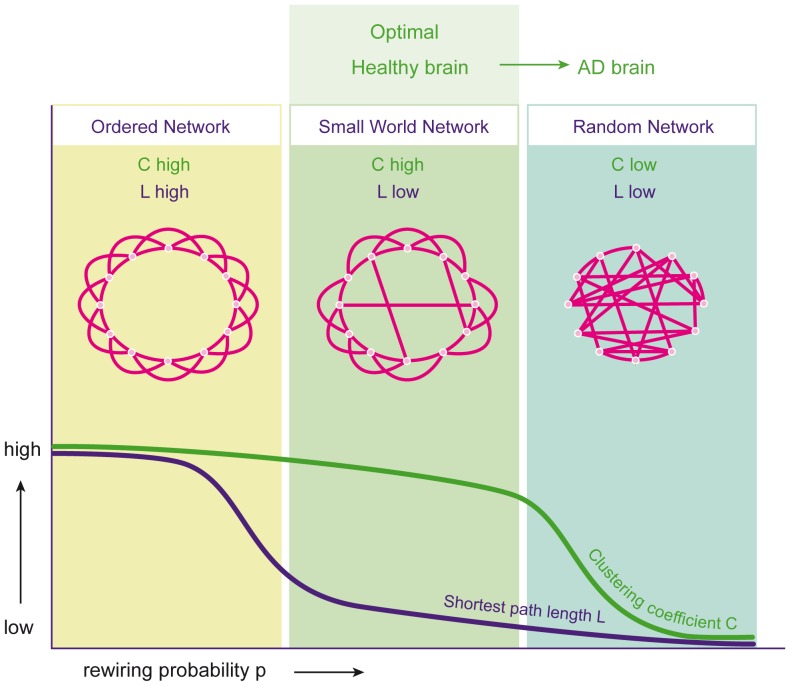
Network models based on clustering coefficient C and path length L. Left: ordered model with high C and high L, middle: small-world model with high C and low L, right: random model with low C and low L. Adapted from Watts and Strogatz, Nature 1998.

One of the major challenges in cognitive studies is to relate cognitive performance to physiological processes, in particular communication in neural networks. Graph theory is a new and effective tool to characterise and quantify the organisation of neuronal networks, and to assess changes due to disease or treatment. Electroencephalography (EEG) is a well-known, widely available and inexpensive tool that reflects synaptic activity directly. Although the exact relationship remains to be clarified, oscillatory activity of neurons measured by EEG is likely to be involved in cognitive processing [11]. Recent advances in the EEG signal analysis allow for the construction of functional networks and have added value in studies of subjects suffering from cognitive problems (for a review see: [12]). EEG changes, assessed with the basic quantitative analysis tools as well as the newer network analysis, have been recognised in AD. These changes consist of a slowing of background activity [13], as opposed to the stable, fast frequency of activity observed in healthy aging [14]. EEG slowing correlates with decreased performance on memory tasks in AD patients [15], [16]. In addition to EEG slowing, changes in EEG functional connectivity (or functional coupling, defined as the statistical interdependence of two or more time series [17]) can be found in AD. Mainly in the faster frequency bands, the coupling between brain regions was found to be lower in AD compared to controls [18]. Additionally, lower functional connectivity has been shown to be correlated with lower scores on cognitive tasks in healthy controls, subjects with mild cognitive impairment, and AD patients [19]–[21]. Based on measures of functional connectivity, so-called graphs can be constructed and analysed. This provides insight into the specific organisation, rather than the strength, of the connections [22], [23]. A graph is an abstract representation of the network (in this case brain activity) with the points in the graph representing the EEG electrodes and the connections representing the functional coupling between points. The organisation of such graphs can be quantified using the theoretical framework of graph theory [24]. The rewiring model of Watts and Strogatz shows how local connectivity and global integration of the networks can be used to assess optimal network structure [25] (Figure 1). The graph-theoretical measure ‘clustering coefficient C’ indicates the amount of interconnectedness of neighbouring points (local connectivity) and has a high value in an ordered network (Figure 1, left) and a low value in a random network (Figure 1, right). The measure ‘path length L’ is a measure of how efficient signals traverse the network (global connectivity, integration, or efficiency). In an ordered network, it takes many steps to reach the other side of the network (high path length value, Figure 1, left); in a random network, it only takes a few steps (low path length value, Figure 1, right). In between the ordered and random network is the small-world network, combining high local connectivity with short path length (Figure 1, middle).

Importantly, many real life networks have been shown to display a small-world configuration, including the human brain [26], [27]. Apparently, brain connectivity is not random, but has an optimal balance between local clustering and global integration. Functional network studies have demonstrated that the optimal small-world network is disrupted in AD patients compared to controls and is reconfigured in a disorganised random topology (Figure 1, right) [18], [28].

Souvenaid® (Nutricia N.V., Zoetermeer, The Netherlands), containing the specific nutrient combination Fortasyn® Connect, has been designed to enhance synapse formation and function in AD compared to control product without the specific nutrient [29], [30]. Souvenaid is a product intended as a medical food for oral consumption under medical supervision with the purpose of addressing disease-specific nutrient requirements. A medical food is a food formulated for enteral intake by patients, taken under physician supervision, and intended to meet the specific nutritional requirements identified for a disease or condition, which cannot be met by modification of the normal diet. The specific nutrient combination Fortasyn Connect comprises docosahexaenoic acid (DHA), eicosapentaenoic acid (EPA), uridine-mono-phosphate (UMP), choline, phospholipids, folic acid, vitamins B6, B12, C, E, and selenium, which are the precursors and cofactors for the formation of neuronal membranes. Preclinical studies have shown that administration of DHA, EPA, UMP and choline can increase phosphatide synthesis [31], neurite outgrowth [32], the number of dendritic spines and the levels of pre- or post-synaptic proteins [33]. Other nutrients, such as B vitamins, act as cofactors by increasing the availability of membrane precursors or by directly affecting the neuronal membrane or membrane synthesis [34], [35]. In a 12-week double-blind, randomised controlled trial in drug-naïve patients with mild AD (“Souvenir I”), improved memory performance was demonstrated after intervention with Souvenaid compared to a control product [9]. These findings were confirmed and extended in the 24-week “Souvenir II” randomised controlled trial in drug-naïve patients with mild AD [36]. Additionally, quantitative EEG effects were seen in the Souvenir II study. Patients receiving Souvenaid had preserved peak frequency and higher functional connectivity in the delta band. The EEG findings support the underlying hypothesis of enhanced synaptic activity as a result of Souvenaid use and suggest a relationship between synaptic changes and memory improvement.

Although Souvenaid increases the formation of neuronal membranes and synaptogenesis in preclinical studies, the effects on the synapse density in humans cannot be investigated directly. Large-scale functional brain network organisation derived from EEG activity can be regarded as a surrogate for macroscopic neuronal communication. It potentially serves as a bridge between mechanistic basic science and clinical outcome. The aim of the present pre-specified secondary analyses of the Souvenir II study was to investigate the effect of Souvenaid on large-scale functional brain network organisation in patients with mild AD by applying network analysis to brain EEG activity. It was hypothesised that Souvenaid maintains or improves the functional brain network organisation in contrast to a shift from an optimal network organisation towards disorganization in the control AD group.

## Methods

The protocol for this trial and supporting CONSORT checklist are available as supporting information; see Checklist S1 and Protocol S1. There were no deviations from the trial protocol.

### 2.1 Ethics Statement

The ethical review boards of each participating centre reviewed and approved the trial protocol (Medisch Ethische Toetsings commissie VUmc, Amsterdam; Medisch Ethische Commissie azM/UM, Maastricht; Commissie Mensgebonden Onderzoek regio Arnhem-Nijmegen; Medisch Ethische Toetsingscommissie Catharina Ziekenhuis, Eindhoven; Medisch Ethische Toetsingscommissie Amphia, Breda; Wetenschapsbureau Jeroen Bosch Ziekenhuis, Den Bosch; LAWO Orbis Medisch Centrum [Lokale Adviesgroep Wetenschappelijk Onderzoek], Sittard; Regionale Toetsingscommissie Patiëntgebonden Onderzoek, Leeuwarden; Medisch Ethische Toetsingscommissie Tergooiziekenhuizen, Blaricum; Commissie Medische Ethiek ZNA/O.C.M.W., Antwerp; Comité voor Medische Ethiek, Sint-Andriesziekenhuis Tielt; Medische Ethische Commissie H. Hartziekenhuis, Roeselare; Ethisch Comité vzw Emmaüs, AZ Sint-Maarten, Mechelen; Ethisch Comité Sint-Trudoziekenhuis, Sint-Truiden; Ethik-Kommission der Universität Ulm; Ethik-Kommission der Ärztekammer Nordrhein; Ethik-Kommission der sächsischen Landesärztekammer; Ethikkommission der medizinischen Fakultät Heidelberg; Ethik-Kommission der Bayerischen Landesärztekammer; Comité ético de investigación clínica. Hospital de la Santa Creu i Sant Pau, Barcelona; Comité ético de investigación clínica. Hospital Clínico San Carlos, Madrid; H.Clinic I Provincial EC Agencia de Ensayos Clínicos, Hospital Clinic de Barcelona; Hospital Virgen Arrixaca EC, Murcia; Comitato Etico Fondazione Ospedale Maggiore, Milan; Comitato Etico Aziende Sanitarie Umbria; Comitato Etico dellâ’Azienda Ospedaliera Universitaria s. Martino di Genova and CPP sud-ouest, Toulouse).

The study was conducted in accordance with the Declaration of Helsinki and the International Conference on Harmonisation-Good Clinical Practice guidelines as appropriate for nutritional products and legislation of the country in which the research was conducted.

### 2.2 Patients

Patient data from the Souvenir II study were used for this manuscript. The Souvenir II study was a 24-week, randomised, controlled, double-blind, parallel-group, multi-country study. The detailed methodology and primary results of the study have been described previously [36]. Briefly, 259 patients ≥50 years of age with a diagnosis of probable AD and a Mini Mental State Examination (MMSE) of ≥20 were recruited and randomised 1∶1 to receive once daily either the active (Souvenaid) or iso-caloric control product as a 125 ml drink for 24 weeks. Duration of subject inclusion was approximately 19 months. The first subject was included in the study 12 November 2009. The last subject completed the study on 30 May 2011. Memory performance measured with a Neuropsychological Test Battery (NTB) was the primary outcome of the study; EEG was studied as a secondary outcome. EEG data were used for the present manuscript and were assessed at baseline, 12, and 24 weeks. The Dutch Trial Registration number for the Souvenir II study is NTR1975. All patients and caregivers gave written informed consent to use their data for research purposes.

### 2.3 EEG Recording

All EEGs were recorded using digital equipment at the local study sites from 19 electrodes at the positions of the 10–20 system: Fp2, Fp1, F8, F7, F4, F3, T4, T3, C4, C3, T6, T5, P4, P3, O2, O1, Fz, Cz, Pz. Sample frequency was at least 200 Hz and at most 512 Hz, depending on local EEG settings. Higher sample frequencies were down-sampled to allow for comparison of consecutive EEGs within patients. A-D precision was 12 bit or higher. Electrode impedance was kept below 5 kOhm. EEG data was filtered online using a high pass filter of 0.16 Hz and a low pass filter of 70 Hz. All EEG data were re-referenced off-line to average reference. At the sites, EEG data were converted to ASCII files and sent to the central reading site at VU University Medical Center for quality check, further preprocessing and analyses (Brainwave software, version 0.9.37, developed by CS; freely available at: home.kpn.nl/stam7883/brainwave.html). From every EEG, four 4096 sample epochs of artifact free data (containing no eye blinks, slow eye movements, excess muscle activity, electrocardiogram artifacts, etc.) were selected by an experienced neurophysiology technician and checked (HW).

### 2.4 EEG Analysis, Functional Connectivity

Functional connectivity between all electrode pairs was established with the Phase Lag Index (PLI), a measure that is relatively insensitive to volume conduction [28]. PLI was calculated in batches, separate for all sample frequencies (200 Hz, 250 Hz, 256 Hz, 400 Hz, 500 Hz and 512 Hz) in four different frequency bands (delta 0.5–4 Hz, theta 4–8 Hz, alpha 8–13 Hz and beta 13–25 Hz) [37].

### 2.5 EEG Analysis, Network Analysis by Graph Theory

**Figure 2:**
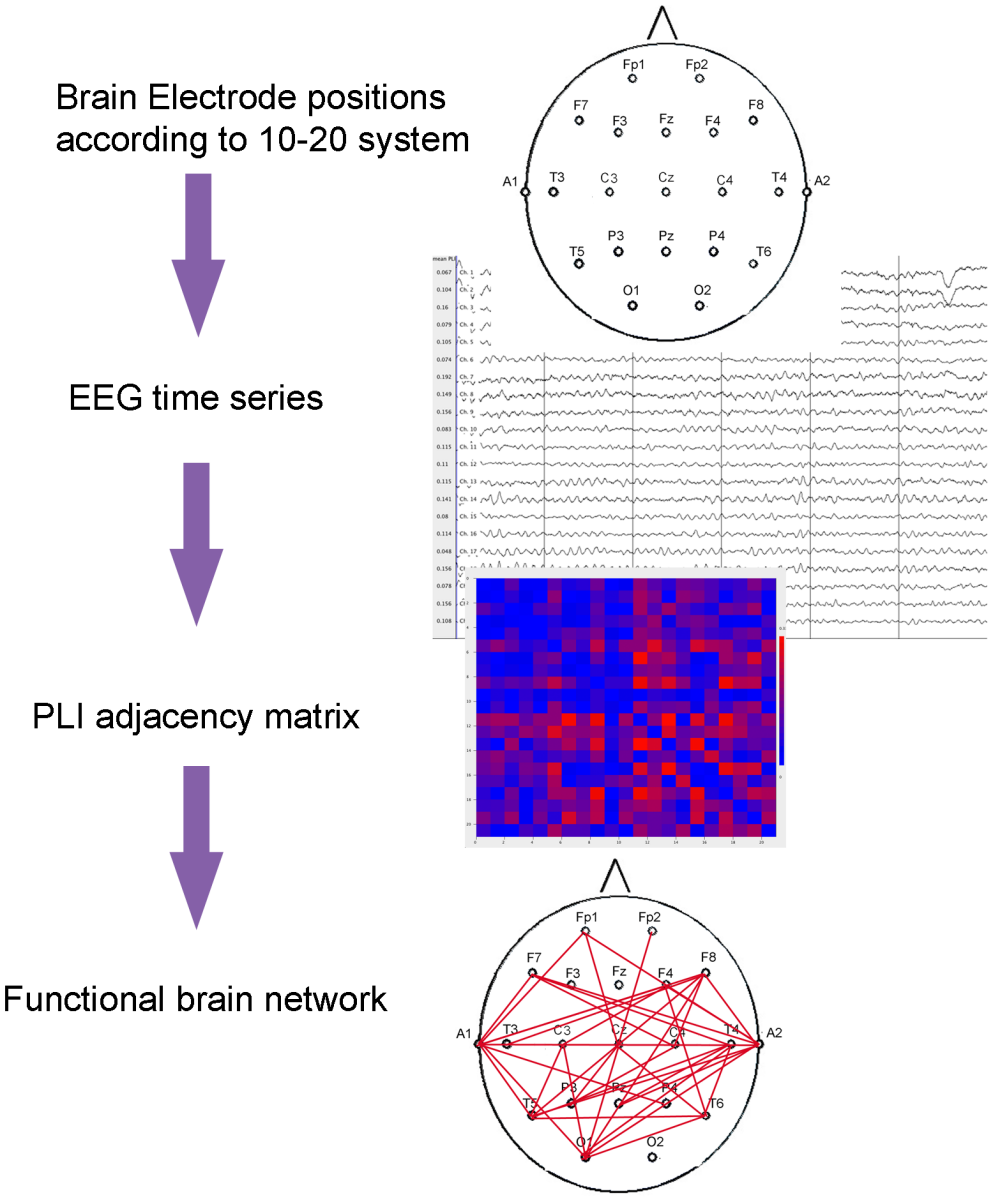
Schematic representation of construction of graphs from EEG time series. EEG time series are measured from scalp electrodes. Phase Lag Index (PLI) as a measure of functional connectivity is calculated between all pairs of electrodes. From the PLI adjacency matrix, the functional brain network is reconstructed and network measures are computed.

In this study pre-specified secondary analyses of the EEG data from the Souvenir II study were conducted. We applied graph theoretical analysis to EEG functional connectivity networks. Figure 2 shows a schematic representation of how graphs are derived from EEG time series.

In this context, the graph represents a brain network: the electrodes are the nodes in the graph; the PLI value for a pair of nodes is the edge weight. From this weighted graph, two basic network measures were computed: the mean weighted clustering coefficient (Cw) and the mean weighted shortest path length (Lw). Cw is defined by the probability that two nodes are connected when they are both connected to the same node (when they ‘share a neighbouring node’), which is a measure of local connectivity (for mathematical definitions used in this study see Stam et al, 2009 [38]). Lw reflects global integration of a network and is defined by the weighted shortest path length, computed according to Dijkstra’s algorithm [24]. Cw and Lw can vary greatly between subjects and are dependent on the size of the graphs and connectivity. In order to compare networks of different subjects, the networks were normalised. This was done by generating 50 random networks for each network by randomly shuffling the PLI values in each adjacency matrix while keeping the matrix symmetry intact. For each ensemble of random networks, the average Cw(<Cw-random>) and Lw (<Lw-random>) were computed. By taking the ratio (network measures of the real network divided by those of random networks), the network measures were normalised for network size and connection strength. The resulting normalised clustering coefficient (gamma, Cw/<Cw-random>), and normalised path length (lambda, Lw/<Lw-random>) were used in further analyses.

### 2.6 Statistical Analysis

EEG data were analysed using mixed models for repeated measures (MMRM) with post-baseline measurements as an outcome, using SAS® software (SAS Enterprise Guide 4.3 for Windows, SAS Institute Inc., Cary, NC, USA). Restricted Maximum Likelihood (REML) was used as an estimation method to estimate the mixed model. Data were normally distributed. In a three-level model with random site-specific intercepts and random subject-specific intercepts, group (treatment arm), time (treatment duration), and the group*time interaction were tested and adjusted for baseline values and sample frequency (baseline and sample frequency were included as covariates). Since EEG data at baseline were missing for 20% of the patients, for the baseline as covariate the centre’s mean baseline value was imputed in case of missing baseline. The two degrees of freedom (df) contrast describing the difference in trajectories over time between active and control groups was taken as the primary indication of treatment effect during the intervention period. In addition, endpoint contrasts were reported. Primary analysis was intention-to-treat (ITT). A per-protocol (PP) analysis was also conducted, excluding subjects with protocol deviations (e.g. violating the eligibility criteria, study product compliance <80%, etc).

To explore the relationship between memory performance and the statistically significant EEG network measures per time point, flexible regression models were used. At baseline, active and control groups were combined together; whereas for 12 and 24 weeks, the groups were analysed separately. To explore the relationship between memory performance and significant EEG measures over time, a random effect mixed model analysis was conducted per group, using the data for each group combined over three time points. In the mixed model the suggested relationship (i.e. quadratic) from the flexible regression models was employed and compared with linear effect.

## Results

### 3.1 Patients

**Figure 3:**
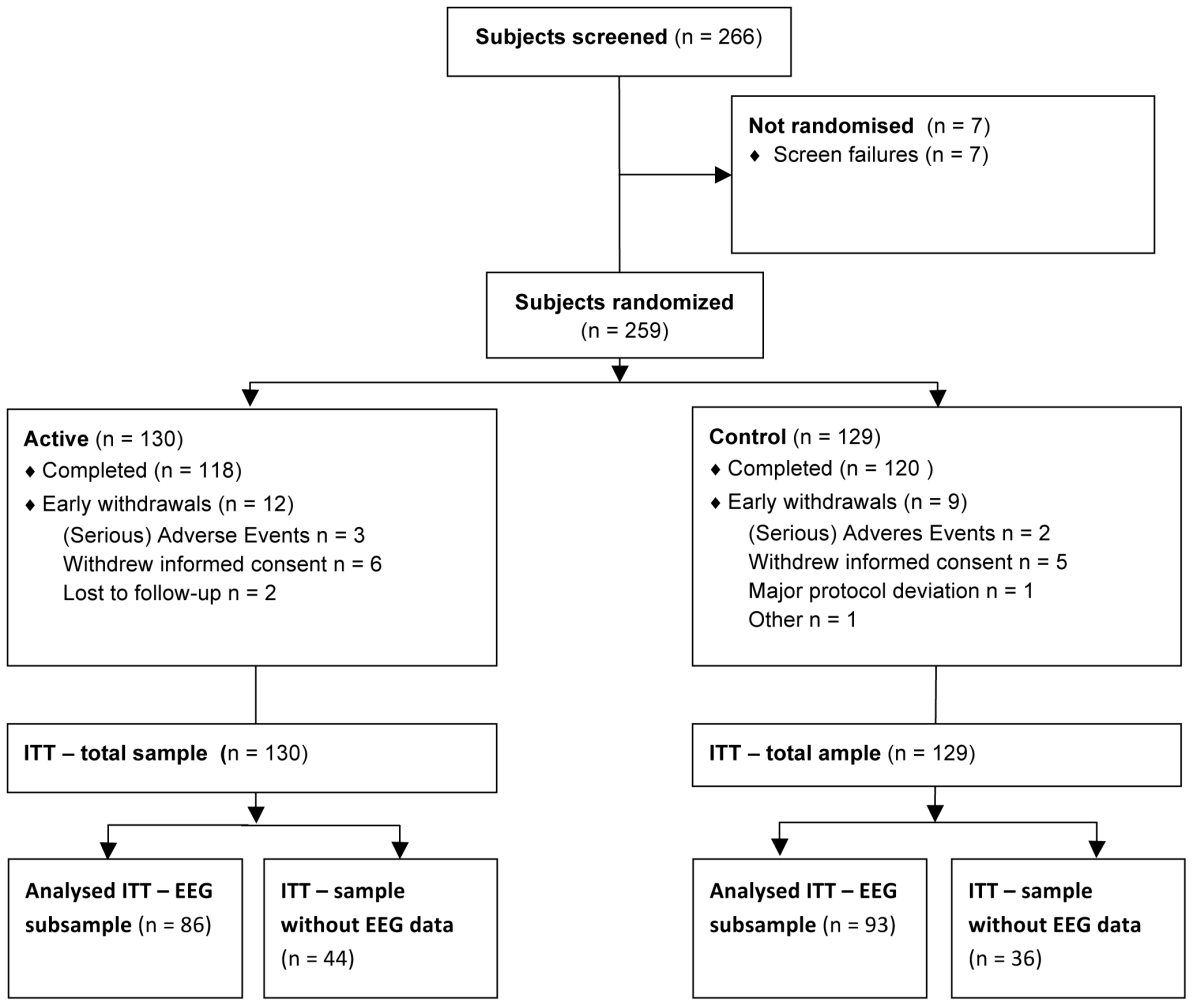
Patient disposition for EEG subsample.

In total, 259 patients were randomised to intervention between November 2009 and May 2011 (see Figure 3). EEG data were available for a subset of 179 subjects (i.e. ITT population; 86 subjects from the active group and 93 subjects from the control group; Table 1), as not all study sites were able to collect high quality EEG data. At 20 sites EEG data were collected. EEG was recorded from on average nine (varying from 3–21) subjects per site. For the mixed model analyses, small sites were pooled, yielding nine (combined) sites with on average 19.9 (varying from 16–24) subjects per (combined) site. Twelve (6.7%) of the subjects who underwent EEG discontinued the study. Protocol deviations occurred in 14 of 179 subjects (10 subjects from the control group and four subjects from the active group): Ten subjects had an average study product compliance of <80% or unknown study product compliance, two subjects received vitamin B12 injections during the study; one subject stopped use of memantine 2 instead of 3 months prior to baseline, and one subject used an antipsychotic at baseline. All these subjects were analysed in the primary ITT analysis, but excluded from the PP analysis. Baseline demographics and characteristics of the subpopulation studied in the EEG analysis are presented in Table 2. The groups did not significantly differ with respect to all characteristics, including age, duration of AD since diagnosis, years of education, baseline MMSE score, gender, and APOE ε4 genotype.

**Table 1:** Number of patients with correct EEG data (intent-to-treat population.

	Control	Active
Baseline	77	66
Week 12	76	73
Week 24	75	70

**Table 2:** Baseline demographics and characteristics of the subset of subjects for whom EEG data were available (intent-to-treat population).

	Control (n = 93)	Active (n = 86)
Male, N (%)	47 (50.5)	45 (52.3)
Age, y [range]	72.5 (8.0) [52–85]	74.1 (6.8) [55–87]
Years of education beyond primary school	6.7 (4.8)	6.7 (4.9)
Duration of AD since diagnosis, months, median [range]	2.0 [0.0–88.0]	1.0 [0.0–38.0]
ApoE ε4 carrier, N (%)		
No	40 (47.6)	39 (48.8)
Yes	44 (52.4)	41 (51.3)
Unknown	9	6
Total MMSE score	25.4 (2.7)	25.1 (2.9)

*Note.* AD: Alzheimer’s disease; MMSE: Mini-Mental State Examination; Data are presented as mean (standard deviation) unless stated otherwise.

Compliance with the study product was very high for both groups. The calculated patient reported compliance during 24 weeks was 96.7% in the control group and 97.2% in the active group, with no difference between study groups (*t*-test, *p* = 0.609).

### 3.2 Network Analysis

**Figure 4:**
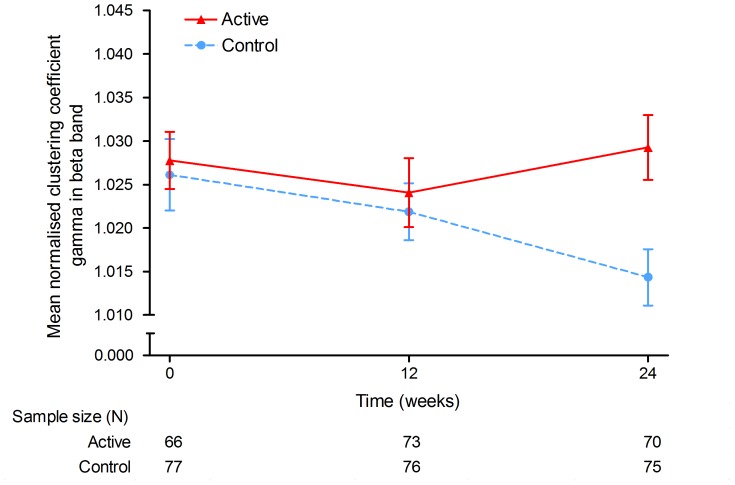
Local clustering of AD functional networks. The normalised clustering coefficient gamma in the beta band during 24-weeks intervention was significantly different between the groups. Blue, dotted line: control product; red, solid line: Souvenaid. X-axis: time (weeks), y-axis: gamma in beta band. Error bars represent standard errors of the mean.

Gamma (the clustering coefficient normalised for network size and connectivity), showed significant differences between active and control groups in the beta band during the 24–week intervention period (mixed models for repeated measures [MMRM], F(2,279) = 4.69, p = 0.010; intraclass correlation coefficient [ICC] = 0.141) (See Table 3 and Figure 4). Additionally, the difference between groups was significant at the 24-week endpoint (MMRM, t(279) = 3.05, p = 0.003).

**Table 3:** Descriptive statistics for the normalised clustering coefficient gamma (intent-to-treat population).

	Control	Active	P-value^1^
**Delta (0.5–4 Hz)**			
Baseline	1.031 (0.036) [77]	1.023 (0.031)	
Week 12	1.031 (0.039) [76]	1.033 (0.037)	
Week 24	1.028 (0.032) [75]	1.037 (0.039)	0.249
24-week trajectory			**0.514**
**Theta (4–8 Hz)**			
Baseline	1.035 (0.036) [77]	1.031 (0.030)	
Week 12	1.035 (0.030) [76]	1.032 (0.033)	
Week 24	1.028 (0.034) [75]	1.033 (0.034)	0.333
24-week trajectory			**0.411**
**Alpha (8–13 Hz)**			
Baseline	1.046 (0.044) [77]	1.050 (0.043)	
Week 12	1.045 (0.045) [76]	1.051 (0.051)	
Week 24	1.043 (0.034) [75]	1.046 (0.052)	0.758
24-week trajectory			**0.741**
**Beta (13–25 Hz)**			
Baseline	1.026 (0.036) [77]	1.028 (0.027)	
Week 12	1.022 (0.028) [76]	1.024 (0.034)	
Week 24	1.014 (0.028) [75]	1.029 (0.031)	0.003
24-week trajectory			**0.010**

*Note.* Data are means (standard deviation).

1 *P*-values were based on a mixed model for repeated measures (2 degrees of freedom contrast) with post-baseline measurements as an outcome.

Figure 4 shows that during the first 12 weeks of intervention, gamma decreased slightly in both groups. The control group showed a further decrease of gamma up to week 24, whereas gamma in the active group remained stable. No significant effect was found in the other frequency bands. The results of the PP analyses in which 14 subjects with protocol deviations were excluded did not change the results.

**Figure 5:**
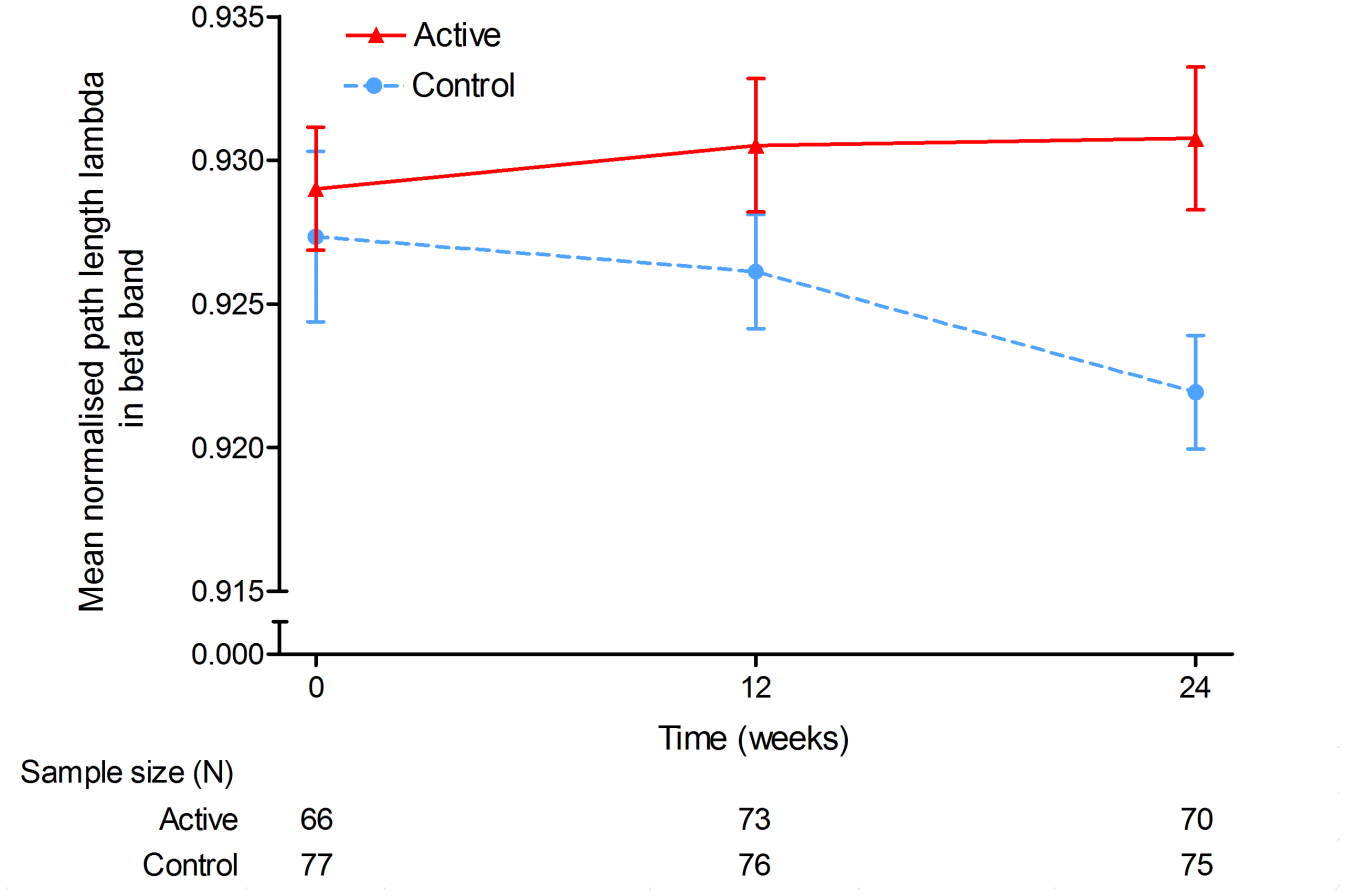
Global integration of AD functional networks. The normalised path length lambda in the beta band during 24-weeks intervention was significantly different between the groups. Blue, dotted line: control product; red, solid line: Souvenaid. X-axis: time (weeks), y-axis: lambda in beta band. Error bars represent standard errors of the mean.

Lambda (the path length normalised for network size and connectivity), as a measure of global network integration, showed a trend for a difference in trajectories over the 24-week study period in the beta band (MMRM, F(2,279) = 2.97, p = 0.053; ICC = 0.083) (See Table 4 and Figure 5). The difference between groups was further confirmed by a significant difference between active and control groups at the 24-week endpoint (MMRM, t(279) = 2.35, p = 0.019). Figure 5 suggests that lambda remained stable in the active group and decreased in the control group, mainly in the second part of the study (week 12 to 24). No significant effects for lambda were observed in the other frequency bands. The results of the PP analyses were similar to the results from the primary ITT analysis.

**Table 4:** Descriptive statistics for the normalised path length lambda (intent-to-treat population).

	Control	Active	P-value^1^
**Delta (0.5–4 Hz)**			
Baseline	0.930 (0.021) [77]	0.924 (0.019)	
Week 12	0.925 (0.021) [76]	0.930 (0.025)	
Week 24	0.925 (0.020) [75]	0.928 (0.018)	0.536
24-week trajectory			**0.600**
**Theta (4–8 Hz)**			
Baseline	0.933 (0.026) [77]	0.933 (0.021)	
Week 12	0.933 (0.022) [76]	0.936 (0.031)	
Week 24	0.933 (0.021) [75]	0.933 (0.033)	0.810
24-week trajectory			**0.635**
**Alpha (8–13 Hz)**			
Baseline	0.946 (0.039) [77]	0.943 (0.034)	
Week 12	0.948 (0.037) [76]	0.943 (0.025)	
Week 24	0.948 (0.031) [75]	0.941 (0.038)	0.269
24-week trajectory			**0.358**
**Beta (13–25 Hz)**			
Baseline	0.927 (0.026) [77]	0.929 (0.017)	
Week 12	0.926 (0.017) [76]	0.931 (0.020)	
Week 24	0.922 (0.017) [75]	0.931 (0.021)	0.019
24-week trajectory			**0.053**

*Note.* Data are means (standard deviation).

1 *P*-values were based on a mixed model for repeated measures (2 degrees of freedom contrast) with post-baseline measurements as an outcome.

### 3.3 Exploratory Analysis: Relationship between Memory Performance and Significant EEG Network Measures

Using flexible regression models per time point, a significant nonlinear relationship between gamma in the beta band and memory performance was observed at 12 weeks in the active group (χ^2^ =  1.14, p = 0.028), indicating a quadratic relationship between gamma in the beta band and memory performance. This significant relationship was not observed at the other time points (Baseline, 24 weeks) or in the control group. The mixed model analysis did not reveal a significant quadratic or linear relationship between memory performance and gamma in the beta band when data of different time points was combined.

For the relationship between lambda in the beta band and memory performance, no significant associations were found using flexible regression models per time point or mixed model analysis using data combined over the time points.

## Discussion

By using EEG as a bridge between bench and bedside, we aimed to investigate the effect of Souvenaid on the macroscopical organisation of electrical brain activity in vivo. Souvenaid has an effect on synaptic integrity and function at the mechanistic basic level [29], and on memory at the clinical level [9], [36]. We hypothesised that the effect of Souvenaid on the synapses also results in improved organisation of brain activity on a larger scale, because memory, such as other cognitive processes, is thought to arise from organisation of communication between brain regions [39].

The present secondary analysis of EEG data from the 24-week Souvenir II randomised controlled trial demonstrated that the functional brain network organisation of patients with mild AD receiving Souvenaid differed significantly from mild AD patients receiving control product. More specifically, the combination of decreasing local clustering and decreasing path length during the 24 weeks of the study in the control group indicated deterioration from a small-world to random network type, according to the pattern seen in AD [40], [41]. The active group remained closer to an optimal network organisation with stable network values. As far as we are aware, we are the first to report a decline of EEG network measures in patients with mild AD receiving control product in a longitudinal study of 24 weeks duration. This points in the same direction as previous cross-sectional research on networks of AD patients [18], [28]. Additionally, a decline in peak frequency of EEG activity was previously found in the control group of this study [36], as expected in progressive AD. These findings indicate that the EEG measures of the control group were representative for the natural course of AD, that they deteriorate in the course of several months in the mild stage, and that these EEG markers can be used to evaluate interventions in this patient group. In the group of patients receiving Souvenaid, the values for optimal small-world network organisation did not decrease, but remained stable during the course of the study in the active group. The direction of these results is in line with the Watts and Strogatz model and with the previous finding of preserved EEG peak frequency in the active group of this study [36].

EEG reflects neuronal communication. It directly measures electrical activity, and the signal at the EEG electrode is formed by the sum of excitatory and inhibitory post synaptic potentials (EPSP and IPSP respectively) in cortical, mainly pyramidal, neurons. EEG oscillations usually are analysed in frequency bands. The basis for this is the notion that oscillations with similar frequencies are involved in the same processes [42]. The relationships between frequency of oscillations and cognitive functions are largely unknown. High frequency oscillations, including beta band activity, are thought to mediate local cortico-cortical connectivity and are associated with several cognitive processes, such as memory. Slower oscillations arise from longer-distance connections between cortical and subcortical areas. The mechanisms underlying the predilection of the intervention effect for the beta band network organisation remain unknown. Several studies have shown an increase in beta band interactions during processes that involve endogenously stimulated processes in for example search strategies and attention [43]. Furthermore, beta band activity has been shown to play a role in maintaining the current cognitive state [43]. We found significant network effects of Souvenaid in the beta band, which may support the biological basis of the effect of Souvenaid on memory.

The clinical implications of the results lie in the importance of structure of brain activity. It has become clear that higher order brain function, such as cognition, is a result of both specialisation and integration [39]. The complex organisational structure of the brain can be measured with advanced signal analysis and network and complex systems theory. In AD, the functional organisation is disrupted, although the cellular mechanisms causing the large-scale network changes are not clear. This study provides information on the link between the properties at synaptic level and macroscopic functional brain organisation: an intervention aimed at the synapses was found to influence overall brain network organisation. Network structure may be regarded as an intermediate step between connected neurons and memory, but the exact relationship of EEG network measures to cognitive or memory performance is not clear. Results from exploratory association analyses failed to show a consistent relationship between memory performance and gamma and lambda in the beta band in this study. We hypothesise that other patient factors influence this complex relationship. Moreover, the changes over time might differ between the network measures and memory. It can be conceived that in AD, mechanisms are in play that constantly optimise the wiring in the network to counteract the effects of the disease and to preserve cognition as long as possible. This will result in a decoupling of network changes and cognition in a progressive disease, such as AD. Buldu et al. supports this hypothesis: the researchers found network changes before the onset of dementia [44]. In addition, it is known that other imaging modalities show changes before cognitive deficits are present [45]. Lastly, EEG is not suitable for the analysis of gamma band activity [46], which may be an important frequency band for cognition [47]. Although these factors restrict detailed analysis into the relationship between functional brain networks and cognition, it is important to gain better understanding how cognition emerges from brain activity. In addition, EEG can only be used as a surrogate endpoint (as opposed to a secondary outcome measure, as used in our study) when cognition can be predicted from EEG. Future research could extend on the current findings by studying the contribution of potential influencing factors in longitudinal studies of longer duration.

Our results raise the question how a presumed non-selective process at the synaptic level can result in changes in organisation at the macroscopical level. The findings in preclinical studies showed an effect of membrane precursors on synaptogenesis: an increase in dendritic spine density and neuritic outgrowth was found after nutrient enrichment [48]. Additionally, an effect on synaptic function was reported, assessed with neurotransmitter concentrations, as well as an effect on behaviour, learning, and memory [49], [50]. We suggest that in addition to the effects of Fortasyn Connect on the synapse in the preclinical studies [29], Souvenaid, compared to the control product, enhances synaptic formation and function in patients with mild AD. The pathological effects of AD are not equally distributed across the brain and there are indications that especially brain areas that are central in the functional network, such as the precuneus and the posterior part of the cingular gyrus, are affected [51], [52]. It is hypothesized that when functional connectivity is enhanced (such as aimed for by Souvenaid), the process of hub failure is counteracted by preserving connectivity outside hub areas, resulting in preservation of the small-world network organisation. Alternatively, the electrically (and possibly also metabolically) more active hub regions might benefit more from Souvenaid, and the effect of the intervention may vary between brain regions. Further studies are needed including higher spatial resolution functional studies to test these hypotheses.

A couple of methodological issues have to be considered. The multicentre design of this trial poses a limitation for the EEG results. EEG quality varied over sites. However, despite the variability in EEGs between sites we do find significant effects, reflecting the robustness of the observed effects. Additionally, the connectivity measure used to construct the networks influences the outcome of the network parameters. The PLI as a measure of connectivity is relatively strict in comparison to other measures like Synchronisation Likelihood and coherence, which makes it a less sensitive but more reliable measure of true functional connectivity. Since all phase differences between EEG time series that cannot reliably be distinguished from volume conduction (zero and II) were disregarded, probably a proportion of genuine functional connections were left out of the analysis. This might have influenced results, and could be one of the reasons that an effect in the alpha band, which is reported to be involved in network changes in AD, could not be established. However, using the PLI and therefore a more specific measure, the connections that were found were more probably real connections. As opposed to the afore mentioned risk of a type II error (false negative results), also the risk of a type I error should be mentioned. We tested two network parameters in four frequency bands, where a false positive result cannot be ruled out. However, the fact that a consistent difference was found between the patient groups across the two network parameters is indicative of a real effect in our opinion.

This study has implications for further research. Group differences in EEG quantitative and network analyses were found during a 24-week intervention period. For a mild AD patient group, this period is therefore sufficient to detect deterioration in the control group. Our study may serve as reference to other intervention studies using EEG as an outcome parameter to examine whether the intervention affects disease-related brain pathology. It is a minimally invasive, patient friendly, low-cost brain imaging modality, and therefore practically feasibly in large multicenter studies. Although EEG has a high temporal resolution, it has a low spatial resolution, compared to magnetoencephalography (MEG) and functional MRI. With higher spatial resolution methods it is possible to study the influence of Souvenaid on other network characteristics, known to be affected by AD, like modularity [53] and hubs in the brain.

It might be more beneficial to introduce nutritional interventions early in the disease, instead of later when the effects of the disease are too extensive to counteract. In patients with mild cognitive impairment (MCI), a shift has been shown towards a more random network configuration [44]. De Haan et al. reported an increase in activity just before rapid degradation of the spiking activity in hub regions, suggesting inhibition with excess activity precedes deactivation in case of impending disconnection [54]. This increase in activity can be seen as an early stage in a progressive disease process, which might coincide with preclinical AD. Intervention studies on cognition and EEG activity in this stage of AD are warranted, given the information that changes in functional connectivity are already present before the dementia syndrome has become obvious.

In conclusion, this study shows that a new approach to Alzheimer’s disease in its early stages (the medical food Souvenaid) by addressing a novel target (synapse formation and function) was found to have an effect on organisation of brain activity as assessed with a novel strategy (EEG-based functional network analysis). In addition, this novel strategy of EEG analysis has been shown to have favourable properties with respect to data acquisition, and therefore a feasible marker of brain activity in multicentre clinical trials. At the same time, it helps to support and further develop hypotheses on the disease process and effects of interventions. The relationship between EEG-based functional networks and cognition is complex and needs further research. The preserved functional EEG network organisation observed in the group receiving Souvenaid strengthens the hypothesis that Souvenaid enhances synaptic formation and function compared to control and has a biological effect on the brains of patients with mild AD.

## Supporting Information

Checklist S1 **CONSORT checklist.**
(DOC)

Protocol S1 **Protocol Sub-study of the Souvenir II study: magnetoencephalography Version 1.3.**
(PDF)
