# Supplementary material for: The Effect of Souvenaid on Functional Brain Network Organisation in Patients with Mild Alzheimer’s Disease: A Randomised Controlled Study
Source: PLoS One. 2014 Jan 27;9(1):e86558. doi: 10.1371/journal.pone.0086558 (PMC3903587; doi:10.1371/journal.pone.0086558)
Supplement: Protocol S1 — Protocol Sub-study of the Souvenir II study: magnetoencephalography Version 1.3. (PDF) [file pone.0086558.s002.pdf]

Protocol

Sub-study of the Souvenir II study: Magnetoencephalography

An exploratory randomised, controlled, double-blind, parallel-group multi-centre study to assess the effect of a medical food on magnetoencephalogram in patients with mild Alzheimer's Disease (AD), a sub-study of the Souvenir II study, protocol Alz.1.C/D

Version : 1.3, based on Local amendment 3

Date : 02 February 2012

Protocol number : Alz.1.C/E

Coordinating investigators: Prof. Dr. Ph. Scheltens
Prof. Dr. C.J. Stam
VU University Medical Center
Amsterdam, The Netherlands

Contact details Sponsor : M.J. van der Mooren, MD, PhD, MSc
Danone Research
PO Box 7005
6700 CA Wageningen
The Netherlands
Tel. +31-317-467800
Fax. +31-317-466500

Version history

Version	Date	Reason for creating new version
1.0	14 April 2009	Document creation
1.1	3 June 2010	Local Protocol Amendment 1
1.2	20 December 2010	Local Protocol Amendment 2
1.3	02 February 2012	Local Protocol Amendment 3

Ethics Statement

This study will be conducted according to the protocol and in compliance with Good Clinical Practice, the ethical principles stated in the Declaration of Helsinki, and other applicable regulatory requirements.

Confidentiality Statement

The information provided in this document is the property of Danone Research, and is shared with you and your staff in confidence. This information should not be disclosed to others without written authorization from Danone Research, except to the extent necessary to ensure adequate conduct of the trial.

PROTOCOL SIGNATURE PAGE - SPONSOR

**Protocol details**	
Study title:	An exploratory randomised, controlled, double-blind, parallel-group, multi-centre study to assess the effect of a medical food on magnetoencephalogram in patients with mild Alzheimer's Disease (AD), a sub-study of the Souvenir II study, protocol Alz.1.C/D
Study name/acronym:	Sub-study of the Souvenir II study - Magnetoencephalography
Protocol number:	Alz.1.C/E

Protocol approved by the sponsor:
We, the undersigned, have reviewed and approved this protocol including the appendices.

	**Director Research Neurology**	**Director Clinical Studies Platform**	**Statistician**	**Medical Affairs Manager**
Name	P.J.G.H. Kamphuis, PhD	M.J. van der Mooren, MD, PhD, MSc	S.H.N. Swinkels, PhD	C.W. Verduyn, MD
Signature				
Date				

PROTOCOL SIGNATURE PAGE - INVESTIGATOR

Protocol approved by the investigator:
I, the undersigned, have reviewed this protocol including the appendices and I agree to the following:

- To conduct the clinical study in compliance with the protocol, GCP, and any other regulatory requirements.
- To obtain protocol approval from an independent Ethics Committee and to comply with their requirements for ongoing review and reporting.
- To comply with procedures for data recording and reporting
- To permit monitoring, auditing and inspection by the sponsor and relevant regulatory agencies.
- To retain study related documents according to regulatory requirements and as agreed with the sponsor.

	**Investigator**
Name:	
Function:	
Signature:	
Date:	

PROTOCOL SYNOPSIS

STUDY TITLE	An exploratory randomised, controlled, double-blind, parallel-group, multi-centre study to assess the effect of a medical food on magnetoencephalogram in patients with mild Alzheimer's Disease (AD), a sub-study of the Souvenir II study, protocol Alz.1.C/D
STUDY NAME	Souvenir II - Magnetoencephalography
PROTOCOL NUMBER	Alz.1.C/E
STUDY PRODUCT(S)	The investigational product, Souvenaid®, is a 125 ml (125 kcal) once-a-day multi-nutrient drink. Souvenaid® contains Fortasyn™ Connect, a specific combination of nutrients, and is intended as a Food for Special Medical Purposes (FSMP) for the dietary management of Alzheimer's Disease (AD). The control product is iso-caloric, similar in flavour, appearance, and composition without Fortasyn™ Connect.
STUDY PHASE	Phase III
STUDY OBJECTIVES	To explore the effect of a medical food on magnetoencephalogram during 24 weeks' intake of investigational product compared to control product in patients with mild AD.<br><br>To explore the effect of a medical food on electroencephalogram, cognition, blood chemistry, tolerance and safety during 24 weeks' intake of investigational product compared to control product in patients with mild AD.
STUDY DESIGN	A 24-weeks, randomised, controlled, double-blind, parallel-group trial.
STUDY DIAGRAM	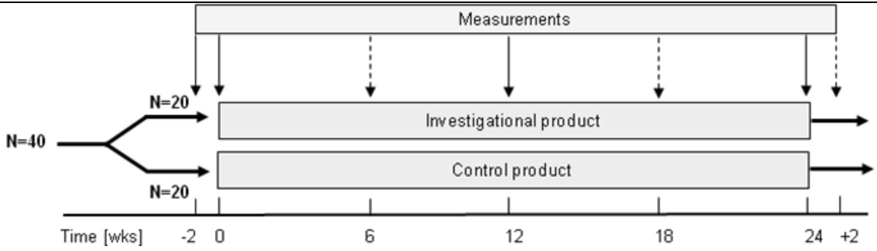
SUBJECTS	20 mild AD patients of the Souvenir II study were enrolled in the MEG sub-study: twelve at the VU Medical Centre and eight at study centres in Madrid and Barcelona. The enrolment of patients will continue until 40 patients have completed all MEG measurements at the VU Medical Centre.
INCLUSION CRITERIA	- Diagnosis of probable AD according to the NINCDS-ADRDA criteria<br>- MMSE score ≥ 20<br>- MRI or CT scan within two years before baseline showing no evidence of any other potential cause of dementia other than AD<br>- Age ≥ 50 years<br>- Availability of responsible caregiver<br>- Written informed consent of patient and caregiver
EXCLUSION CRITERIA	- Diagnosis of significant neurological disease other than AD, including vascular dementia according to NINDS-AIREN criteria, cerebral tumour, Huntington's Disease, Parkinson's Disease, normal pressure hydrocephalus, seizures, and other entities<br>- Use within 3 months prior to baseline, or expected need during the study of donepezil, rivastigmine, galantamine, and/ or memantine<br>- Geriatric Depression Scale > 6 on 15-item scale

	- Use within two months prior to baseline of:<br>- omega-3 fatty acid containing supplements<br>- oily fish (when consumed more than twice a week)<br>- Use within one month prior to baseline of:<br>- atropine, scopolamine, tolterodine, hyoscyamine, biperiden, benztropine, trihexyphenidyl, oxybutynin<br>- antipsychotics<br>- vitamins B, C and/ or E > 200% RDI<br>- high energy and/ or high protein nutritional supplements/medical foods<br>- other investigational products<br>- Change in dose within one month prior to baseline of:<br>- lipid lowering medication<br>- antidepressants<br>- antihypertensive medication<br>- Alcohol or drug abuse in opinion of the investigator<br>- Institutionalisation in nursing home<br>- Investigator's uncertainty about willingness, ability, or medical status of patient to comply with protocol requirements
RECOMMENDATIONS DURING STUDY	During the entire study, the treating investigator must monitor that the patients do not use:<br>- omega-3 fatty acid containing supplements<br>- oily fish more than twice a week<br>- vitamins B, C and/ or E > 200% RDI<br>- high energy and high protein nutritional supplements/medical foods<br>- other investigational products<br>- donepezil, rivastigmine, galantamine, and/ or memantine<br>During the study, the treating investigator must monitor that patients do not change in dose or type of:<br>- lipid lowering medication<br>- antidepressants<br>- antihypertensive medication<br>- benzodiazepines
STUDY PARAMETERS	- Magnetoencephalogram (MEG)<br>- Electroencephalogram (EEG)<br>- Neuropsychological Task Battery (NTB)<br>- Blood chemistry: blood plasma levels of HCy, vitamin E and fatty acid profile in erythrocyte membrane<br>- Vital signs: pulse and blood pressure<br>- (Serious) adverse events<br>- Liver function (serum ALT, AST, alkaline phosphatase, and gamma GT)<br>- Renal function (serum creatinin)
OTHER ITEMS<br>Screening/ baseline only	- Date of birth<br>- Sex<br>- Ethnicity<br>- Smoking habits<br>- Excessive alcohol consumption<br>- Physical activity<br>- Duration of AD since diagnosis

Throughout study	- Education<br>- Family history of AD<br>- Relevant medical history and coexisting diseases<br>- Use of concomitant medication and nutritional supplements<br>- Blood collection for possible future analysis<br>- Study product compliance
STUDY GROUPS	Patients will be equally randomised to one of the following groups:<br>- receiving the investigational product<br>- receiving the control product
STUDY PRODUCT REGIME	One 125 ml tetra pack of either investigational or control product daily.
STUDY PERIOD	The study duration for each subject is approximately 26 to 28 weeks.
STUDY DESCRIPTION	Patients with AD who appear to fulfil the eligibility criteria are informed about the study and invited for a screening visit. Patients meeting the in- and exclusion criteria are enrolled during the baseline visit. Patients are randomly allocated to either the investigational or control arm for a double-blind period of 24 weeks. At baseline, 12, and 24 weeks the major study parameters are assessed and the study product is dispensed. To monitor safety, to keep patients motivated, and to optimise study product compliance, phone calls are conducted at 6 and 18 weeks. A final follow-up call is done 2 weeks after the last site visit.
STATISTICAL ANALYSIS	A linear mixed model will be used. An interim analysis will be performed on the first 20 patients that completed all MEG measurements at the VU Medical Centre, aiming to recalculate the sample size. Details on the interim analysis will be specified in the Interim Statistical Analysis Plan, which will be finalised before the start of the interim analysis. Details of the final statistical analysis are specified in the Statistical Analysis Plan of the Souvenir II study.
SPONSOR	Danone Research, Wageningen, The Netherlands

TABLE OF CONTENTS

PROTOCOL SIGNATURE PAGE - SPONSOR 2

PROTOCOL SIGNATURE PAGE - INVESTIGATOR 3

PROTOCOL SYNOPSIS 4

TABLE OF CONTENTS 7

ABBREVIATIONS AND DEFINITIONS 10

1 INTRODUCTION 12

2 STUDY OBJECTIVES 13

2.1 STUDY OBJECTIVES 13
2.2 STUDY DESIGN 13
2.3 STUDY DESCRIPTION 13
2.4 SCHEMATIC DIAGRAM OF TRIAL DESIGN 14
2.5 DURATION OF THE STUDY PER PATIENT 14

3 SUBJECTS 14

3.1 STUDY POPULATION 14
3.2 ELIGIBILITY CRITERIA 14
3.2.1 Inclusion criteria 14
3.2.2 Exclusion criteria 14
3.3 SUBJECT RECRUITMENT 15
3.4 RECOMMENDATIONS DURING THE STUDY 15
3.5 PATIENT DISCONTINUATION DURING THE STUDY 16
3.5.1 Criteria for patient discontinuation 16
3.5.2 Procedures in case of patient discontinuation 16
3.6 ROLE OF THE CAREGIVER 16

4 STUDY PARAMETERS 16

4.1 MAGNETOELECTROENCEPHALOGRAM (MEG) 16
4.2 ELECTROENCEPHALOGRAM (EEG) 16
4.3 NEUROPSYCHOLOGICAL TEST BATTERY (NTB) 16
4.4 BLOOD CHEMISTRY 18
4.5 TOLERANCE AND SAFETY PARAMETERS 18
4.6 OTHER ITEMS TO BE COLLECTED 18
4.6.1 Screening/ baseline only 18
4.6.2 Throughout study 19
4.6.3 Study product-related 19

5 STUDY PROCEDURES 20

5.1 PROCEDURES PER VISIT 20

5.1.1 *Screening visit* ... 20
5.1.2 *Baseline visit (within two weeks after Screening visit or combined)* ... 20
5.1.3 *6 and 18 -week phone calls* ... 20
5.1.4 *12-week visit* ... 20
5.1.5 *24-week visit* ... 21
5.1.8 *Follow-up phone call (2 weeks after 24-week visit)* ... 21
5.1.9 *End of study visit (in case of premature termination)* ... 21
5.2 TESTS ... 21
5.3 EEG AND MEG ... 22
5.4 VISIT WINDOWS ... 22

6 STUDY PRODUCT ... 23

6.1 NAME AND DESCRIPTION OF STUDY PRODUCTS ... 23
6.2 SUMMARY OF FINDINGS FROM CLINICAL AND NON-CLINICAL STUDIES ... 23
6.3 BENEFITS AND RISKS ASSESSMENT ... 23
6.4 DESCRIPTION OF ROUTE OF ADMINISTRATION AND DOSAGE ... 23
6.5 PREPARATION, PACKAGING, LABELLING AND STORAGE ... 23
6.5.1 *Preparation and directions for use* ... 23
6.5.2 *Packaging and labelling* ... 23
6.5.3 *Storage conditions* ... 24
6.6 PRODUCT SHIPMENT, ACCOUNTABILITY AND DESTRUCTION ... 24

7 RANDOMISATION AND (UN-)BLINDING ... 24

7.1 RANDOMISATION ... 24
7.2 UNBLINDING PROCEDURE ... 24

8 SAFETY REPORTING ... 25

8.1 DEFINITIONS ... 25
8.1.1 *AE* ... 25
8.1.2 *SAE* ... 25
8.1.3 *SSAR* ... 25
8.1.4 *SUSAR* ... 25
8.2 (SERIOUS) ADVERSE EVENTS HANDLING ... 25
8.2.1 *(S)AE recording* ... 25
8.2.2 *SAE reporting by the investigator* ... 26
8.2.3 *SAE review and reporting by the Sponsor* ... 26
8.2.4 *Follow-up of SAEs* ... 26
8.3 NEW RELEVANT SAFETY INFORMATION ... 26

9 STATISTICS ... 27

9.1 STATISTICAL FORMULATION OF THE STUDY HYPOTHESIS ... 27
9.2 SAMPLE SIZE CALCULATION ... 27
9.3 INTERIM ANALYSIS ... 27
9.4 ANALYSIS SETS ... 28
9.5 DESCRIPTIVE STATISTICS ... 28
9.6 STATISTICAL METHODS FOR DATA ANALYSIS ... 28

9.6.1 Multilevel modelling ... 28
9.6.2 Sensitivity analysis ... 28

10 ETHICAL CONSIDERATIONS ... 28

10.1 BASIC PRINCIPLES AND REGULATIONS ... 28
10.2 ETHICS COMMITTEE ... 28
10.3 INFORMED CONSENT ... 29
10.4 PATIENTS NOT USING ALZHEIMER MEDICATION ... 29
10.5 OPEN-LABEL EXTENSION PERIOD ... 29
10.6 CONFIDENTIALITY OF STUDY DATA ... 29
10.7 INCENTIVES/COMPENSATION FOR PATIENTS ... 29
10.8 INSURANCE ... 30

11 ADMINISTRATIVE ASPECTS AND PUBLICATION ... 30

11.1 STUDY MONITORING AND AUDITS ... 30
11.2 SOURCE DATA ... 30
11.3 DATA HANDLING ... 30
11.4 STORAGE OF DOCUMENTS ... 30
11.5 CRITERIA FOR PREMATURE TERMINATION OF THE TRIAL ... 30
11.6 PUBLICATION POLICY ... 31

REFERENCES ... 32

APPENDIX I SCHEDULE OF ASSESSMENTS ... 35

APPENDIX II NINCDS-ADRDA CRITERIA ... 36

ABBREVIATIONS AND DEFINITIONS

Term	Explanation
AD	Alzheimer's Disease
AE	Adverse Event
ALT	Alanine aminotransferase
aPTT	Activated Partial Thromboplastin Time
AST	Aspartate transaminase
Control product	Product used as a comparison to the investigational product
Coordinating investigator	An investigator assigned the responsibility for the coordination of investigators at different centres participating in a multicentre trial
COWAT	Controlled Word Association Test
CRF	Case Report Form
CRO	Contract Research Organisation
CT	Computed Tomography
D-KEFS	Delis-Kaplan Executive Function Scale
DMC	Data Monitoring Committee
EEG	Electroencephalogram
FSMP	Food for Special Medical Purposes
Gamma GT	Gamma-glutamyltransferase
GCP	Good Clinical Practice
ICH	International Conference on Harmonisation
Investigational product	The product to be tested in the study
Investigator	The person responsible for the conduct of the clinical trial at the trial site
ITT	Intention-to-treat
MEG	Magnetoencephalogram
MMSE	Mini Mental State Examination
MRI	Magnetic Resonance Imaging
NINCDS-ADRDA	National Institute of Neurological and Communicative Disorder and Stroke - Alzheimer's Disease and Related Disorder Association
NINDS-AIREN	National Institute of Neurological Disorders and Stroke and Association Internationale pour la Recherché et l'Enseignement en Neurosciences
NMDA	N-methyl-D-aspartic acid
NTB	Neuropsychological Test Battery
PP	Per-protocol
(Principal) Investigator	Responsible for the conduct of the clinical trial at a trial site. In case of a study team, the investigator is the responsible leader of the team and may be called the principal investigator
PT	Prothrombin Time
RAVLT	Rey Auditory Verbal Learning Test
RDI	Recommended Daily Intake
SAE	Serious Adverse Event
Study product	Product to be used, comprising the investigational and control product

SUSAR	Suspected Unexpected Serious Adverse Reaction
TMT	Trail Making Test
WMS	Wechsler Memory Scale

1 INTRODUCTION

Alzheimer's Disease (AD) is a progressive neurodegenerative disorder with a multifactorial pathophysiology, of which the exact cause is still unknown. To date, there is no satisfactory treatment for AD. Current pharmaceutical treatments like the acetylcholinesterase inhibitors donepezil, rivastigmine, galantamine, and the N-methyl-D-aspartic acid (NMDA) receptor antagonist memantine, usually provide a temporary reduction of symptoms and are not free of side-effects. This illustrates the strong unmet need for effective therapeutic interventions. The potential role of nutrition in such strategies is rapidly gaining interest.

Scientific research indicates a clear link between nutrition and AD. Nutritional subclinical deficiencies are frequently reported among patients with AD [1-4]. Levels of nutrients such as some fatty acids, vitamins and micronutrients, have been found too low. This is either due to reduced daily consumption, or to increased need throughout the disease [4, 5]. On the other hand, the intake of specific ingredients, e.g. omega-3 fatty acids and vitamins is associated with a reduced risk of developing AD [1, 6-13]. Furthermore, it has been reported that intake of nutritional components can be protective (e.g. Mediterranean diet, fish consumption) [14].

Several nutrients such as omega-3 fatty acids, choline and certain vitamins, are considered essential for the brain, since they act as precursors in the synthesis pathway of phosphatides, the building blocks of membranes. Preclinical studies have demonstrated that combinations of specific nutrients synergistically enhanced phosphatide formation and improve synapse formation. Multi-nutrients have shown to increase (synaptic) membrane synthesis and thereby to enable increased neurite outgrowth and dendritic spine density in preclinical studies [15, 16]. Further, these nutrients improve neurotransmitter release and performance on hippocampus-dependent cognitive functions in test animals [17, 18]. By providing specific nutrients, membrane integrity can also be improved, and thereby membrane-dependent processes such as amyloid precursor protein processing can be beneficially influenced [19]

Based on the evidence (see also Product Information Brochure), a multi-nutrient intervention has been formulated to improve the quantity and quality of neuronal membranes, to stimulate synapse formation, and to enhance the functions of membrane-bound proteins in patients with Alzheimer's Disease. This intervention addresses the specific nutritional needs in AD patients. It is developed for the dietary management of AD, and intended to be used as a Food for Special Medical Purposes (FSMP).

Preclinical evidence shows improved synapse formation after supplementation with nutrients present in the investigational product as measured by: increased neurite outgrowth [20], increased dendritic spine density and increased levels of synaptic proteins in brain homogenates [21-23]. In humans it is not possible to quantify synaptic density directly. However, the electrical activity generated by neuronal communication at the synapse can be measured indirectly by so-called 'functional connectivity' studies using electroencephalography (EEG) or magneto-encephalography (MEG) and can serve as a potential biomarker for the effect of the study product on synapses [24]. MEG is a very detailed (151 channels compared to 21 with routine EEG) method to study brain activity and neuronal network organization. MEG provides functional mapping information and is thereby able to image neurological function. A big advantage of MEG over EEG is that the signal is not disrupted by intervening tissues like the skull and that it does

not require a reference. Together, these features offer a more detailed study into the mode of action of the study product. Like the EEG signal, the MEG signal is also divided into different frequency bands for purposes of analysis.

Numbers of synapses have been shown to be reduced in AD patients [25, 26] and studies with EEG have demonstrated reductions in brain electrical activity and loss of connectivity and organization of neuronal networks [27-29]. Moreover, MEG recordings in AD patients have shown widespread loss of functional interactions in the alpha and beta bands [30]. However, currently no intervention studies have been performed in AD patients to improve abnormalities seen with EEG or MEG recordings.

The main objective of the current sub-study is to explore whether MEG has the potential to detect effects in brain functional mapping as a result of the study product. This study was started as a sub-study of the Souvenir II study (Alz.1.C/D). By the time that patient enrolment for the Souvenir II study was completed (15 Dec 2010), enrolment for the MEG sub-study as planned (n=40 patients) was not fulfilled yet. At that time 20 patients (out of 40 patients planned) were enrolled in the MEG sub-study, of which 12 patients in the VU Medical Centre.

This amendment protocol includes a prolongation of the MEG sub-study until 40 patients have completed all MEG measurements at the VU Medical Centre. For each discontinued patient, a new patient will be included. It is proposed to incorporate the core items of the Souvenir II study in the MEG sub-study protocol. Eligibility criteria and the design of the study are similar to the original Souvenir II study to ensure that the integrity of this sub-study is not affected. In addition, the number of assessments has been reduced compared to the Souvenir II study protocol in order to limit the burden for the patients, while focusing on the main objectives of the MEG sub-study.

2 STUDY OBJECTIVES

2.1 Study objectives

1. To explore the effect of a medical food on magnetoencephalogram during 24 weeks' intake of investigational product compared to control product in patients with mild AD.
2. To explore the effect of a medical food on electroencephalogram, cognition, blood chemistry, tolerance and safety during 24 weeks' intake of investigational product compared to control product in patients with mild AD.

2.2 Study design

This is a 24-weeks, randomised, controlled, double-blind, parallel-group, trial.

2.3 Study description

Patients with AD who appear to fulfil the eligibility criteria are informed about the study and invited for a screening visit. Patients meeting the in- and exclusion criteria are enrolled during the baseline visit. Patients are randomly allocated to either the investigational or control arm for a double-blind period of 24 weeks. At baseline, 12, and 24 weeks the major study parameters are assessed and study product is dispensed. To monitor safety, to keep patients motivated, and to

optimise study product compliance, phone calls are conducted at 6 and 18 weeks. A final follow-up call is done 2 weeks after the last site visit.

2.4 Schematic diagram of trial design

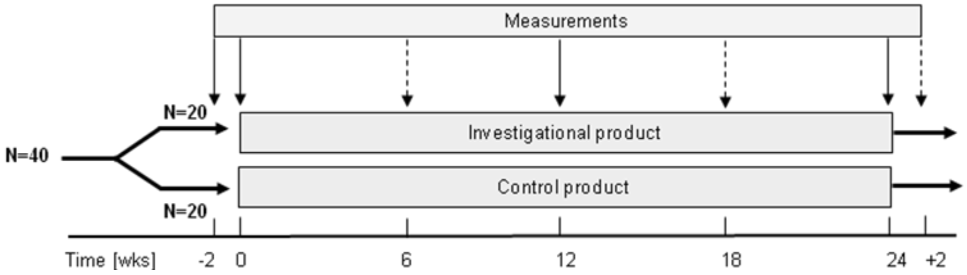

A detailed overview including all visits and phone calls is included in appendix I.

2.5 Duration of the study per patient

The study duration for each patient is approximately 26 to 28 weeks, comprising an optional screening period of up to two weeks and an off-study product follow-up period of two weeks.

3 SUBJECTS

3.1 Study population

20 mild AD patients of the Souvenir II study were enrolled in the MEG sub-study: twelve at the VU Medical Centre and eight at study centres in Madrid and Barcelona. The enrolment of patients will continue until 40 patients have completed all MEG measurements at the VU Medical Centre.

3.2 Eligibility criteria

At the moment of randomisation, the patients must meet the in- and exclusion criteria listed below. At the moment of screening, the patients should either meet the criteria, or it must be reasonably expected that all criteria are fulfilled within two weeks after screening, prior to baseline.

3.2.1 Inclusion criteria

- Diagnosis of probable AD according to the NINCDS-ADRDA criteria
- MMSE score ≥ 20
- MRI or CT scan within two years before baseline showing no evidence of any other potential cause of dementia other than AD
- Age ≥ 50 years
- Availability of responsible caregiver (i.e. living with patient or contact with patient at least 5 days/week of which at least 3 visits/week, capable and willing to assist patient's study product intake, to attend study visits, and to provide input on outcome)
- Written informed consent of patient and caregiver

3.2.2 Exclusion criteria

- Diagnosis of significant neurological disease other than AD, including vascular dementia according to NINDS-AIREN criteria, cerebral tumour, Huntington’s Disease, Parkinson’s Disease,

normal pressure hydrocephalus, seizures, and other entities
- Use within 3 months prior to baseline, or expected need during the study of donepezil, rivastigmine, galantamine, and/ or memantine
- Geriatric Depression Scale > 6 on 15-item scale
- Use within two months prior to baseline of:
  - omega-3 fatty acid containing supplements
  - oily fish (when consumed more than twice a week)
- Use within one month prior to baseline of:
  - atropine, scopolamine, tolterodine, hyoscyamine, biperiden, benztropine, trihexyphenidyl, oxybutynin
  - antipsychotics
  - vitamins B, C and/ or E > 200% RDI
  - high energy and/ or high protein nutritional supplements/medical foods
  - other investigational products
- Change in dose within one month prior to baseline of:
  - lipid lowering medication
  - antidepressants
  - antihypertensive medication
- Alcohol or drug abuse in opinion of the investigator
- Institutionalisation in nursing home
- Investigator's uncertainty about willingness, ability, or medical status of patient to comply with protocol requirements

3.3 Subject recruitment

Patients are recruited by the investigator in the medical centre during their routine visits. Recruitment is facilitated by reviewing medical records by the site staff.

3.4 Recommendations during the study

- During the study, the treating investigator must monitor that the patients do not use:
  - omega-3 fatty acid containing supplements
  - oily fish more than twice a week
  - vitamins B, C and/ or E > 200% RDI
  - high energy and high protein nutritional supplements/medical foods
  - other investigational products

- During the study, the treating investigator must monitor that patients do not change in dose or type of:
  - lipid lowering medication
  - antidepressants
  - antihypertensive medication
  - benzodiazepines

(Change in) use of these medications and supplements will not directly lead to discontinuation, but will be regarded as a protocol deviation. Therapy with the following drugs is reason for study withdrawal: donepezil, rivastigmine, galantamine, and/ or memantine.

3.5 Patient discontinuation during the study

3.5.1 Criteria for patient discontinuation

Patients should terminate the study prematurely in the following situations:

- in case further participation is a health risk for the patient, at supervising clinician's or sponsor's discretion
- in case a patient has decided to resign from further participation in the trial
- In case of the use of donepezil, rivastigmine, galantamine, and/ or memantine (see paragraph 3.4)
- In case of a protocol deviation a patient may be withdrawn from the study at Danone Research's discretion

3.5.2 Procedures in case of patient discontinuation

Any patient who discontinues participation before completion of the study protocol must be reported to and will be reviewed by Danone Research. Also for these patients, the investigator must complete the Case Report Form (CRF) including the clinical summary. Whenever feasible, all scheduled procedures of the end of study visit (see paragraph 5.1.9) should be completed, preferably as soon as possible. Alternative medical care for the discontinued patient is to be arranged by the investigator if necessary. For those patients who discontinued due to the occurrence of adverse events potentially related to the study product, follow-up must be reported until the adverse event has abated, or until a stable situation has been reached, with findings recorded in the CRF.
For each discontinued patient at the VU Medical Centre, a new patient will be included.

3.6 Role of the caregiver

Depending on the autonomy of the patient, the caregiver should assist or supervise the daily intake of the study products and the daily reporting of intake in the diaries. All patients should be accompanied by the caregiver during the visits to the investigator and assist the patient during the telephone calls.

4 STUDY PARAMETERS

4.1 Magnetoelectroencephalogram (MEG)

MEG assessments will be performed at baseline, the 12-week and 24-week visit. Basic frequency analysis and functional connectivity analysis will be conducted.

4.2 Electroencephalogram (EEG)

EEG assessments will be performed at baseline, the 12-week and 24-week visit. Basic frequency analysis and functional connectivity analysis will be conducted.

4.3 Neuropsychological Test Battery (NTB)

The NTB will be administered_at baseline, the 12-week and 24-week visit to assess cognition.
The chosen version is based on the NTB shown to be sensitive in patients with mild AD [31, 32].
The NTB in the current study consists of the following cognitive tasks:

- RAVLT immediate and delayed recall and recognition test
- Wechsler Memory Scale (WMS) verbal paired associates – immediate and delayed recall
- WMS Digit Span
- Trail making tests part A and B
- Category Fluency
- Controlled Word Association Test
- Orientation task of the ADAS-cog
- Letter Digit Substitution Test

Details on the different tests from the NTB are described below:

RAVLT immediate and delayed recall and recognition test
For the Rey Auditory Verbal Learning Test (RAVLT), patients hear a list of 15 words and are asked for their immediate recall. Five trials are administered. Delayed recall of the word list will be examined after an interference period. In the recognition test, unrelated distracting words are intermixed with the words of the list and patients are asked which of the words were read aloud before [33].

WMS Verbal Paired Associates – immediate and delayed recall
In the Verbal Paired Associates test [34], the patient is read a group of eight word pairs. Then, the first word of each pair is read and the patient is asked to supply the second word from memory. This is repeated for three up to six groups of word pairs, dependent on patient’s performance. The score for the immediate recall test ranges from 0-24. After a delay, the test is conducted again for one group of eight word pairs, the first word of each pair is read and the patient is asked to supply the second word again. The score for this delayed recall test ranges from 0-8.

WMS Digit Span
The Digit Span consists of the WMS Digits Forward and Digits Backward tests [34]. In this test, number sequences of increasing length are read to the patient, who is asked to repeat these either forward (Digits Forward) or backward (Digits Backward). The score is derived from the number of sequences correctly recalled, with a range from 0 to 12 for both the forward and backward test.

Trail Making Tests
Both parts of the Trail Making Test (TMT; conditions 2 and 4 of the Delis-Kaplan Executive Function Scale (D-KEFS)) consist of 25 circles distributed over a sheet of paper. In condition 2, the circles include both numbers (1-16) and letters (A-P), and the patient should draw lines to connect the numbers in ascending order as quickly as possible while disregarding the letters. In condition 4, the circles again include both numbers (1-16) and letters (A-P); as in condition 2, the patient draws lines to connect the circles in an ascending pattern, but with the added task of alternating between the numbers and letters (i.e. 1-A-2-B-3-C, etc.) [35-37].

Category Fluency
The Category Fluency (part of the D-KEFS) measures the ability to recollect as many words that belong to a certain category [38], for instance the semantic category ‘animals’. The score is the number of different items belonging to the category named in 60 seconds.

Controlled Word Association Test
The Controlled Word Association Test (COWAT) measures the patient's ability to make verbal associations to specified letters (e.g. C, F and L).

Orientation task of the ADAS-cog
ADAS-cog is the cognitive subscale of the Alzheimer's Disease Assessment Scale (ADAS), which was developed and validated for assessing cognitive and non-cognitive functioning in AD patients [39]. In this trial, the complete ADAS-cog is not included, as the primary focus of this trial is to study the effect on a version of the Neuropsychological Test Battery that is supposed to be sensitive in the early stage of AD [31, 32]. In this trial with mild to very mild AD patients, only the orientation task of the ADAS-cog is used as part of the NTB because this task is deemed to be sensitive for impairment in the early stage of AD. This task is designed to determine how well oriented the patient is with regard to time and place.

Letter Digit Substitution Test
The Letter Digit Substitution Test is a measure for information processing speed [40]. Nine different letters are coupled with nine different numbers in a key. Below this key, a random series of letters in cells is presented, and patients are instructed to write down the corresponding digit to the letters as quickly as possible. The score is the number of correctly substituted numbers in 60 seconds.

4.4 Blood chemistry

Blood plasma levels of homocysteine (HCy), Vitamin E and fatty acid profile in erythrocyte membranes will be measured. Blood is collected, handled, and analysed according to the detailed instructions of the Laboratory Manual as provide by Danone Research.
For all blood parameters (including efficacy and safety), venous blood samples are taken, with a maximum of 30 ml in total per patient for each of the baseline and 24-week visits.

4.5 Tolerance and safety parameters

Parameters on tolerance and safety in this study are:
- Vital signs: pulse and blood pressure are measured using standard clinical equipment.
- (Serious) adverse events (including occurrence of easy bruising, nose bleeding and gastrointestinal tolerance). For definitions and reporting, see chapter 8.
- Blood parameters:
    - Liver function: serum concentrations of alanine aminotransferase (ALT), aspartate transaminase (AST), alkaline phosphatase, and gamma-glutamyltransferase (gamma GT)
    - Renal function: serum concentration of creatinin

Blood is collected, handled, and analysed according to the detailed instructions of the Laboratory Manual as provided by Danone Research.

4.6 Other items to be collected

4.6.1 Screening/ baseline only

Other patient characteristics measured in this study at screening/ baseline only are:
- Date of birth
- Sex
- Ethnicity as categorized into one of following groups:

  - Caucasian/ white (includes people of Mediterranean, European, Hispanic, Middle Eastern origin)
  - Asian (includes people of Chinese, Indian, Pakistani, Bangladeshi, Japanese origin)
  - black (includes people of African descent e.g. African American)
  - combination of previous groups
  - other
- Smoking habits:
  - current use in number of packs (number of cigarettes per day divided by 20)
  - historical use in number of pack years (number of cigarettes per day multiplied by the number of years the patient has smoked divided by 20) and if applicable time since smoking cessation
- Excessive alcohol consumption defined as the occurrence of a two-year episode with an average alcohol intake per week of > 21 drinks for men and > 14 drinks for women:
  - recent/ current: within five years prior to study
  - historical: any time throughout life until five years prior to study
- Physical activity assessment [41]:
  - Question on engagement in regular exercise and if applicable:
  - The frequency (three times a week, once a week, less than weekly) and type of physical activity (more vigorous than walking, walking, less vigorous than walking)
- Duration of AD since diagnosis: will be calculated from date of diagnosis in number of months
- Education as registered in number of years of formal education after finishing primary school as defined per country excluding internships in working/ learning program.
- Family history of AD in number of family members of first degree with history compatible with AD.
- Relevant medical history and coexisting diseases including at least neurological diseases, diabetes, cerebrovascular accidents, severe weight loss, clinically diagnosed depression, head injury, and additionally any relevant diseases or status in opinion of the investigator

4.6.2 Throughout study

- Use of concomitant medication and supplements comprising pre-existing and new use of medication and vitamin and mineral supplements throughout the study, with special attention for lipid-lowering medication, anti-depressants, and anticoagulants.
- Blood biomarkers for possible future analysis to support the biochemical mode of action and markers for nutritional status: inflammatory markers, methylation markers, markers for nutritional status, lipids, vitamins, minerals and trace elements.
- Blood is collected, handled and analysed according to the detailed instructions of the Laboratory Manual as provided by Danone Research. The storage of blood for future analyses will be mentioned explicitly in the informed consent. Blood will be stored for a maximum duration of 5 years at Danone Research.

4.6.3 Study product-related

Study product compliance: study product intake as recorded in a provided diary on a daily basis.

5 STUDY PROCEDURES

5.1 Procedures per visit

Patients who seem eligible are informed in oral and written form about the study and invited for a screening visit.

5.1.1 Screening visit

After signing the informed consent form (see paragraph 10.3), the in- and exclusion criteria (including MMSE score) are checked, and when the patient is considered eligible, following assessments are checked and conducted:

- relevant medical history and coexisting disease
- use of medication (including possible historical use of AD medication) and supplements
- patient characteristics: date of birth, sex, ethnicity, smoking habits, alcohol consumption, physical activity, duration of AD since diagnosis, education, family history of AD
- When there is no MRI or CT-scan from within two years before baseline available, a new MRI or CT-scan is done before the Baseline visit.

5.1.2 Baseline visit (within two weeks after Screening visit or combined)

When the Baseline visit is separate from the Screening visit the in- and exclusion criteria are checked, including the MMSE.
In case all eligibility criteria are met, the patient is randomly allocated to either the investigational or control arm for a period of 24 weeks. Following assessments/ activities are conducted prior to the first study product intake:

- relevant medical history and coexisting diseases
- changes in use of concomitant medication and supplements
- vital signs: pulse and blood pressure
- blood collection for safety and efficacy
- cognition (NTB)
- EEG
- MEG
- instructions for study product storage and intake and diary completion
- dispensing of study product and diary

5.1.3 6 and 18 -week phone calls

To monitor safety, to keep the patients motivated and to optimise study product compliance, the patients are contacted by phone. Following issues are additionally asked for:

- occurrence and resolving of adverse events
- changes in use of concomitant medication and supplements

5.1.4 12-week visit

During this site visit, following issues are asked for or conducted:

- occurrence and resolving of adverse events
- changes in use of concomitant medication and supplements
- cognition (NTB)
- EEG
- MEG

- diary collection and check of consistency with number of tetra packs used and issued
- dispensing of study product and diary

5.1.5 24-week visit

During the last site visit, following issues are asked for or conducted:
- occurrence and resolving of adverse events
- changes in use of concomitant medication and supplements
- vital signs: pulse and blood pressure
- blood collection for safety and efficacy
- cognition (NTB)
- EEG
- MEG
- product return
- diary collection and check on consistency with number of tetra packs used and issued

5.1.8 Follow-up phone call (2 weeks after 24-week visit)

During the final call, following issues are asked for:
- occurrence and resolving of adverse events
- changes in use of medication and supplements

5.1.9 End of study visit (in case of premature termination)

For patients who discontinued study participation before completion of the study protocol, all scheduled procedures of the end of study visit should be completed whenever feasible, preferably as soon as possible. The following issues are asked for or conducted:
- occurrence and resolving of adverse events
- changes in use of concomitant medication and supplements
- vital signs: pulse and blood pressure
- blood collection for safety
- cognition (NTB)
- EEG
- MEG
- product return
- diary collection and check on consistency with number of tetra packs used and issued

For a schematic overview, the Schedule of Assessments is included in Appendix I.

5.2 Tests

Prior to the conduct of the tests, the investigator must ensure that the patient can give reliable input, by questioning on physical and mental issues that could affect the test results, and the use of sedative-hypnotics and/ or anxiolytics. In case the patient has used sedative-hypnotics and/ or anxiolytics in the three days prior to the tests or investigator expects that the tests cannot be completed reliably for other reasons, either the tests should be rescheduled to another date within the visit window (see below), or the test results must remain missing for that study visit.
All study personnel conducting the NTB must be trained and should continue their role throughout the complete duration of the study whenever possible. For the conduct and scoring of the tests in this study, the detailed instructions of the Test Manual in local language as provided by Danone Research must be followed.

5.3 EEG and MEG

Prior to the EEG and MEG measurements at week 12 and week 24, the investigator must verify whether there was - if applicable - any change in the use of benzodiazepines. If there were changes in the use of benzodiazepines in three days prior to the EEG and MEG measurements, the EEG and MEG should be rescheduled to another date within the visit window (see below), or the EEG and MEG must remain missing for that study visit.

5.4 Visit windows

Attempts should be made to schedule visits at the exact date. In case this is not possible, visits should be scheduled within the windows included below:
Baseline visit: within 14 days after Screening visit or combined with Screening at the same day
6-week call: at 42 ± 7 days after Baseline visit
12-week visit: at 84 ± 7 days after Baseline visit
18-week call: at 126 ± 7 days after Baseline visit
24-week visit: at 168 ± 7 days after Baseline visit
End of study visit: as soon as possible after study discontinuation before completion of study protocol
Follow-up call: at 14 ± 7 days after 24-week visit

Regarding the MEG assessments, attempts should be made to schedule the MEG assessments on the same day as the other study assessments. Because of the limited availability of the MEG, the baseline MEG assessment may be conducted within 7 days before or after the other baseline assessments, despite of the fact that patients may have started using study product already. MEG assessments at 12 and 24 weeks should be conducted 84 ± 7 days and 168 ± 7 days after the Baseline visit respectively, according to the visit windows described above.

6 STUDY PRODUCT

6.1 Name and description of study products

The investigational product, Souvenaid®, is a 125ml (125kcal) once-a-day multi-nutrient drink. Souvenaid® contains Fortasyn® Connect, a specific combination of nutrients, and is intended as a FSMP for the dietary management of Alzheimer's Disease (AD). The control product is iso-caloric, similar in flavour, appearance, and composition without containing Fortasyn® Connect.

6.2 Summary of findings from clinical and non-clinical studies

A summary of findings of clinical and preclinical studies is provided in the Product Information Brochure.

6.3 Benefits and risks assessment

From a proof of concept study in 225 AD subjects with the same investigational product, it has been concluded that the investigational product has a good safety profile and is well tolerated throughout 24 weeks of supplementation. The study showed that the product when given for 12 weeks improves memory in mild and very mild AD patients.
Concerns on possible increased risk for bleeding after omega-3 fatty acid supplement use have been reported, albeit on higher dosages than given in the investigational product. However, reviews show that such concerns are unfounded, even for patients treated with antiplatelet and anticoagulant therapies [42, 43]. The dose of omega-3 fatty acids in this study is below the maximum level which is 'generally recognised as safe' by the USA Food and Drug Administration. Of the procedures, venepuncture for collecting blood samples might cause bruising. For the description of the investigational product, see also the Product Information Brochure.

6.4 Description of route of administration and dosage

Study products are intended for oral use. Patients consume one 125 ml tetra pack of study product per day.

6.5 Preparation, packaging, labelling and storage

6.5.1 Preparation and directions for use

The study product, comprising both the investigational and control product, is ready-to-drink. It should preferably be served chilled and shaken before use, drunk from the tetra pack using the attached straw, and consumed with breakfast. Once opened, the product should be consumed within one hour. Any unused contents should be discarded thereafter.

6.5.2 Packaging and labelling

The tetra packs containing study product are packaged in trays of 30. Complete or partial trays are distributed, depending on the planning of product transport to the patient and the patient's flavour preference. It is recommended to provide equal numbers of flavours for the first study period. Tetra packs and boxes are labelled in accordance with applicable laws and regulations in such a way that the double-blind design of the study is effectively maintained throughout the study. Labels on the investigational as well as the control product contain information required for regulatory as well as identification purposes.

6.5.3 Storage conditions

The investigator is responsible for storing the study product in a secure, limited access storage area. The study product should be stored at room temperature, protected from extremes of light and humidity.

6.6 Product shipment, accountability and destruction

At every shipment of study product to the site, a letter of receipt is included. Upon receipt, the investigators must check the delivered study products on completeness and possible damages, and fax the completed letter of receipt to the Danone Research contact person. Product dispensing and return at the site is recorded on an accountability log. Throughout the study, the product accountability must be documented per individual patient and per flavour on a product accountability log. At the end of the study, study products will be either destroyed on site with adequate documentation, or returned to Danone Research for destruction.

7 RANDOMISATION AND (UN-)BLINDING

7.1 Randomisation

For the prolonged sub-study, patients are randomly assigned in a 1:1 ratio to either of the two study groups: investigational group (n=10) or control group (n=10). Determination of whether a patient will receive the active product or the control product will be based on a randomisation list, developed using a computer random number generator by Danone Research. Four different randomisation codes will be used for randomisation (E, F, G, H); two letters per product. The details of the randomisation sequence will be unknown to the investigator and study centre staff, and will be contained in a set of opaque sealed envelopes, each bearing on the outside only the name of the study centre and a number. After inclusion of a subject in the study, the appropriate numbered envelope will be opened at the study centre by the responsible person (i.e. investigator, trial nurse), the letter inside will tell whether the subject will receive a product with code E, F, G, or H.

7.2 Unblinding procedure

At each study centre, one sealed envelope containing four sealed envelopes with the randomisation codes is available. Only in the case of a medical emergency, the investigator is authorized to break the code for a specific patient. Following unblinding procedures must be followed:

- during working hours (Monday-Friday 8.30 AM - 5 PM CET): the Danone Research contact person must be contacted before breaking the code
- outside working hours: the Danone Research contact person must be informed as soon as possible after unblinding

In both situations, the reason for breaking the code must be recorded both on the CRF and on the code envelope, including signature and date of the investigator who opened it. At the end of the study, the (sealed) randomisation envelopes must be returned to Danone Research.

8 SAFETY REPORTING

8.1 Definitions

8.1.1 AE

An Adverse Event (AE) is any untoward medical occurrence in a patient or clinical investigation subject administered an investigational product and which does not necessarily have a causal relationship with this treatment (ICH-GCP definition).

8.1.2 SAE

A Serious Adverse Event (SAE) is any untoward medical occurrence or effect that at any dose:
- results in death
- is life-threatening (at the time of the event)
- requires hospitalisation or prolongation of existing inpatients' hospitalisation
- results in persistent or significant disability or incapacity
- is a congenital anomaly or birth defect

8.1.3 SSAR

A Suspected Serious Adverse Reaction (SSAR) is both:
- an SAE that is judged to be at least possibly related to the study product by either the investigator or sponsor, and
- expected (listed in the Product Information Brochure and/or in paragraph 6.3 of this protocol)

8.1.4 SUSAR

A Suspected Unexpected Serious Adverse Reaction (SUSAR) is both:
- an SAE that is judged to be at least possibly related to the study product by the either investigator or sponsor, and
- unexpected (not listed in the Product Information Brochure or in paragraph 6.3 of this protocol)

8.2 (Serious) Adverse Events handling

8.2.1 (S)AE recording

Any (S)AE as reported spontaneously by the patient or observed by the investigator or staff, is recorded on the AE in the CRF during the course of the study. The investigator must ensure that this information, including onset, duration and nature of event, severity, and action taken, is captured. SAEs must additionally be recorded on the SAE report provided by Danone Research.

The severity of any (S)AE is scored as follows:

Mild:	transient or mild discomfort; no medical intervention/therapy required
Moderate:	mild to moderate limitation in activity; some assistance may be needed; and/or minimal medical intervention/therapy required
Severe:	marked limitation in activity; some assistance usually required; and/or significant medical intervention/therapy/hospitalisation required

The relationship of the (S)AE to the study product is assessed as being not related/unlikely/ possibly/probably/ definitely related.

8.2.2 SAE reporting by the investigator

The investigator must fax the completed SAE report to Danone Research. The initial report must be sent within 48 hours (2 working days) after first notice, the follow-up report as soon as possible.

8.2.3 SAE review and reporting by the Sponsor

Danone Research must review all reported SAEs. In case the SAE is a potential SUSAR, the Medical Monitor must decide whether unblinding is required.
Danone Research must report all SAEs to the accredited Ethics Committee that approved the protocol. SAEs are reported annually as line listings or according to the requirements of the Ethics Committee, SUSARs are reported within 7 (fatal and life threatening events) or 15 days (other events).

8.2.4 Follow-up of SAEs

All SAEs are followed-up by the investigator until they have abated, or until a stable situation has been reached. Depending on the event, follow-up may require additional tests or medical procedures as indicated, and/ or referral to the general physician or a medical specialist.

8.3 New relevant safety information

The investigator must inform the patients and the reviewing accredited ethics committee if anything occurs that may negatively affect the burden or risks of participation as foreseen in the research proposal. The study may be suspended pending further review by the accredited ethics committee, provided that suspension does not jeopardise the patients' health. The investigator will take care that all patients are kept informed.

9 STATISTICS

9.1 Statistical formulation of the study hypothesis

The hypothesis formulation for this study is:

H_0: the effect of using investigational product is equal to the effect of using control product on the MEG in mild AD patients

H_1: the effect of using investigational product is unequal to the effect of using control product on the MEG in mild AD patients

9.2 Sample size calculation

This MEG sub-study is an exploratory study and as far as known, no literature on intervention studies in AD patients to improve abnormalities seen with MEG recordings is known where the sample size calculation could be based on. No formal sample size calculation was therefore performed. In order to gain insight into the effect of the study product on the MEG assessments, a total number of 40 patients will be invited to participate in this sub-study, of which 20 patients in this prolonged sub-study. With the proposed sample size of 20 patients per group we will be able to detect a treatment difference of about 1 standard deviation (effect size 1) with a power of 90% using t-test and a two-tailed alpha of 0.05.

9.3 Interim analysis

An interim analysis will be performed for the first 20 patients that completed all MEG measurements at the VU Medical Centre, aiming to recalculate the sample size. To this end, both an estimate of the nuisance and an estimate of the treatment effect size for the exploratory MEG parameters will be calculated using the intention to treat data set of patients enrolled at the VU Medical Centre.

To our knowledge, there is no information available in literature that can be used to decide which MEG parameter(s) should be considered for the interim analysis. Therefore, the main outcome parameter that will be used for the interim analysis will depend on the outcome of the secondary EEG data from the Souvenir II study and will be described in the Interim Statistical Analysis Plan.

A specific interim data file will be created for the interim analysis. The interim data file contains the required variables only. Subject identification numbers will be recoded and all other variables that could lead to subject identification will be deleted. The Clinical Study Supplies Manager will provide the statistician with the X-Y group identification (for codes E, F, G, H). A record from the interim data file, containing the X-Y group identification, will be difficult to match to individual subjects in the ongoing study.

A Data Monitoring Committee (DMC) will be installed to evaluate the interim analysis. After evaluation of the interim analysis, the statistician and the DMC members will have no further involvement in the study. Based on the results of the interim analysis, an upward adaptation of the sample size may be suggested (> 40 completed patients at the VU Medical Centre). If the sample size calculation based on the interim estimates suggests that less than n=40 will already give sufficient power, the sample size will remain n=40. Since only upward adaptation of the sample size is allowed, the significance level does not need to be adjusted. No further protocol changes can be based on this interim analysis.

Details on the interim analysis will be specified in the Interim Statistical Analysis Plan, which will be finalised before the start of the interim analysis.

9.4 Analysis sets

The primary analyses on the outcome parameters will be intention-to-treat (ITT) analysis. Additional per-protocol analyses will be carried out. Definitions on the ITT and PP data sets will be described in detail in the Statistical Analysis Plan, which will be finalised before unblinding of the study.

9.5 Descriptive statistics

Baseline data will be described and summarized either by means and standard deviations or medians and interquartile ranges as appropriate for continuous data, or by number and percentages for categorical data.

9.6 Statistical methods for data analysis

9.6.1 Multilevel modelling

A linear mixed model will be used with intervention group as fixed factor. Outcome parameters with a continuous distribution will be analyzed using linear models. Outcome parameters with discrete distribution (dichotomous variables, ordered categorical variables, and counts) will be analyzed with generalized linear models. Transformations may be used to improve distribution. If the distribution of an outcome parameter clearly deviates from a standard distribution non parametric tests are used. Details on the statistical analysis will be documented in the Statistical Analysis Plan prior to database lock.

9.6.2 Sensitivity analysis

The sensitivity of the conclusions of the study to the choice of covariates included or to the choice of the relationship between covariates and outcome will be investigated. Adjusted estimates of the intervention effect will be compared to unadjusted estimates.

10 ETHICAL CONSIDERATIONS

10.1 Basic principles and regulations

The investigator must ensure that this study is conducted in full conformance with the principles of the ‘World Medical Association Declaration of Helsinki’ (59th WMA General Assembly, Seoul, October 2008), International Conference on Harmonisation (ICH) guidelines for Good Clinical Practice (GCP) as appropriate for nutritional products, and local legislation of the country in which the research is conducted, whichever affords the greater protection to the participants.

10.2 Ethics committee

This protocol, the patient information and informed consent, and any material as requested, are submitted to the applicable ethics committee by the investigator according to local legislation. Approval from the ethics committee must be obtained before starting the study, and should be documented in a letter to the investigator specifying the date on which the ethics committee met and granted the approval, the composition of the ethics committee, and version and date of all submitted documents.
The investigator must submit a status report at least annually to the ethics committee that approved the protocol.

10.3 Informed Consent

Prior to study enrolment, written informed consent must be obtained from each patient and caregiver. If a patient is unable to decide upon participation him- or herself, the patient's legally acceptable representative must sign the informed consent form.
It is the responsibility of the investigator to obtain the written informed consent after adequate explanation to the patient and/or the patient's legally acceptable representative –if applicable- of the aims, methods, source of funding, the anticipated benefits and potential risks of the study and the discomfort it may entail. Written informed consent must be obtained prior to screening, with the understanding that consent may be withdrawn at any time without prejudice. Two copies of each informed consent are signed: one is given to the signer and one is retained in the Investigator Site File on site.

10.4 Patients not using Alzheimer medication

A recent 12-week proof of concept study with a similarly designed optional 12-week extension period in 225 drug naïve mild AD patients [44] demonstrated that the investigational product improved 12-week memory performance. Furthermore, this proof of concept study showed that the investigational product has a good safety profile and is well tolerated throughout 24 weeks of supplementation. The investigator had the possibility to prescribe medical therapy for Alzheimer's Disease at any time during the study, which would lead to discontinuation from the study. However, from the patients completing the 12-week study, 85% percent entered the extension period and 81% completed the 24-week study [45]. This indicates that 24-weeks of withholding existing Alzheimer medication did not result in any concerns and was considered ethically acceptable.

Like for the proof of concept study mentioned above, for the current study the investigator has the possibility to prescribe medical therapy for Alzheimer's disease at any time during the study. This is however reason for withdrawal (see paragraph 3.4).

10.5 Open-label extension period

Patients who have completed the study will be offered to continue in an open-label extension period, in which they will receive the investigational active product. Details on the open-label extension period are described in a separate protocol.

10.6 Confidentiality of study data

The investigator is responsible for treating patient and study information as confidential. The investigator should ensure that the patient information will not be made publicly available. All patient study records are identified by the patient identification number to maintain patients' confidentiality. In order to avoid reducibility to individuals, fictitious patient initials will be collected. Identification codes lists that link the patients' names to the patients' identification number must be stored in the Investigator Site File.

10.7 Incentives/Compensation for patients

Travel and parking costs are reimbursed to patients participating in the trial.

10.8 Insurance

Danone Research has affected an insurance covering damage to patients through injury or death caused by the study product and/or study procedures, and liability insurance, both according to local legislation.

11 ADMINISTRATIVE ASPECTS AND PUBLICATION

11.1 Study monitoring and audits

Study monitoring under responsibility of or by Danone Research staff is performed through various stages of the trial. Monitoring includes on-site visits to assure that the investigation is conducted according to the protocol and in order to comply with applicable regulations and guidelines. On-site review of CRFs includes the review of forms for completeness, clarity, and consistency with source documents available for each patient.

The investigator must permit trial-related monitoring visits, audits, review by the ethics committee and regulatory inspections, and allow direct access to source data and source documents provided that patient confidentiality is protected. In case of an audit appointed by Danone Research, the investigator will receive timely written notification in advance.

11.2 Source data

All source data are captured in the patient’s files.

11.3 Data handling

Data are collected by means of a CRF and other data collection forms (e.g. patient diary). Danone Research is responsible for designing and provision of these data collection tools. The investigator should file all data per patient. An explanation for the omission of any required data should appear on paper data collection tools. Danone Research receives CRF data and collects data collection forms from the sites and performs data management according to the Standard Operating Procedures. In case of queries, the investigator should respond within the time windows as agreed upon.

11.4 Storage of documents

Danone Research provides the investigator with an Investigator Site File. The investigator is responsible to keep this Investigator Site File updated and available for inspection. Study documents are not destroyed without prior written agreement between Danone Research and the investigator. Should the investigator wish to assign study documents to another party, or move them to another location, Danone Research must be notified first. (Copies of) all documents pertaining to the conduct of the trial must be kept stored by the investigator for a period of at least 15 years.

11.5 Criteria for premature termination of the trial

Both Danone Research and the investigator reserve the right to discontinue the trial at any time as defined in the Clinical Trial Agreement. Should this be necessary, the procedures will be arranged after review and consultation by both parties. In terminating the trial, Danone Research

and the investigator will assure that adequate consideration is given to the protection of the interests of all patients. The investigator must notify the ethics committee of the premature termination.

11.6 Publication policy

Danone Research acknowledges its responsibility to publish and disseminate results of scientific interest arising from this trial. Danone Research and investigator(s) will mutually agree on the publication policy in the Clinical Trial Agreement. This trial will be registered in a public clinical trial database.

REFERENCES

1. Conquer, J.A., et al., *Fatty acid analysis of blood plasma of patients with Alzheimer's disease, other types of dementia, and cognitive impairment.* Lipids, 2000. **35**(12): p. 1305-12.
2. Charlton, K.E., et al., *Lowered plasma vitamin C, but not vitamin E, concentrations in dementia patients.* J Nutr Health Aging, 2004. **8**(2): p. 99-107.
3. Rinaldi, P., et al., *Plasma antioxidants are similarly depleted in mild cognitive impairment and in Alzheimer's disease.* Neurobiol Aging, 2003. **24**(7): p. 915-9.
4. Clarke, R., et al., *Folate, vitamin B12, and serum total homocysteine levels in confirmed Alzheimer disease.* Arch Neurol, 1998. **55**(11): p. 1449-55.
5. Adunsky, A., et al., *Plasma homocysteine levels and cognitive status in long-term stay geriatric patients: a cross-sectional study.* Arch Gerontol Geriatr, 2005. **40**(2): p. 129-38.
6. Morris, M.C., et al., *Consumption of fish and n-3 fatty acids and risk of incident Alzheimer disease.* Arch Neurol, 2003. **60**(7): p. 940-6.
7. Tully, A.M., et al., *Low serum cholesteryl ester-docosahexaenoic acid levels in Alzheimer's disease: a case-control study.* Br J Nutr, 2003. **89**(4): p. 483-9.
8. Barberger-Gateau, P., et al., *Fish, meat, and risk of dementia: cohort study.* Bmj, 2002. **325**(7370): p. 932-3.
9. Kyle, D.J., et al., *Low serum docosahexaenoic acid is a significant risk factor for Alzheimer's dementia.* Lipids, 1999. **34 Suppl**: p. S245.
10. Kalmijn, S., et al., *Dietary fat intake and the risk of incident dementia in the Rotterdam Study.* Ann Neurol, 1997b. **42**(5): p. 776-82.
11. Zandi, P.P., et al., *Reduced risk of Alzheimer disease in users of antioxidant vitamin supplements: the Cache County Study.* Arch Neurol, 2004. **61**(1): p. 82-8.
12. Engelhart, M.J., et al., *Dietary intake of antioxidants and risk of Alzheimer disease.* Jama, 2002. **287**(24): p. 3223-9.
13. van Gelder, B.M., et al., *Fish consumption, n-3 fatty acids, and subsequent 5-y cognitive decline in elderly men: the Zutphen Elderly Study.* Am J Clin Nutr, 2007. **85**(4): p. 1142-7.
14. Barberger-Gateau, P., et al., *Dietary patterns and risk of dementia: the Three-City cohort study.* Neurology, 2007. **69**(20): p. 1921-30.
15. Wang, L., et al., *Dietary uridine-5'-monophosphate supplementation increases potassium-evoked dopamine release and promotes neurite outgrowth in aged rats.* J Mol Neurosci, 2005. **27**(1): p. 137-45.
16. Wurtman, R.J., et al., *Synaptic proteins and phospholipids are increased in gerbil brain by administering uridine plus docosahexaenoic acid orally.* Brain Res, 2006. **1088**(1): p. 83-92.
17. De Bruin, N.M., et al., *Combined uridine and choline administration improves cognitive deficits in spontaneously hypertensive rats.* Neurobiol Learn Mem, 2003. **80**(1): p. 63-79.
18. Holguin, S., et al., *Chronic administration of DHA and UMP improves the impaired memory of environmentally impoverished rats.* Behav Brain Res, 2008. **191**(1): p. 11-6.
19. Broersen, L.M., et al. *Reduced Beta-Amyloid Plaque Burden And Neurodegeneration In APP/PS1 Transgenic Mice Following Multi-Nutrient Dietary Intervention*. in *ICAD*. 2008.
20. Pooler, A.M., et al., *Uridine enhances neurite outgrowth in nerve growth factor-differentiated PC12 [corrected].* Neuroscience, 2005. **134**(1): p. 207-14.
21. Sakamoto, T., M. Cansev, and R.J. Wurtman, *Oral supplementation with docosahexaenoic acid and uridine-5'-monophosphate increases dendritic spine density in adult gerbil hippocampus.* Brain Res, 2007.
22. Cansev, M. and R.J. Wurtman, *Chronic administration of docosahexaenoic acid or eicosapentaenoic acid, but not arachidonic acid, alone or in combination with uridine, increases brain phosphatide and synaptic protein levels in gerbils.* Neuroscience, 2007.

23. Cansev, M., et al., *Oral administration of circulating precursors for membrane phosphatides can promote the synthesis of new brain synapses.* Alzheimers Dement, 2008. **4**(1 Suppl 1): p. S153-68.
24. Ramakers, G.J., *Neuronal network formation in human cerebral cortex.* Prog Brain Res, 2005. **147**: p. 1-14.
25. Scheff, S.W., et al., *Hippocampal synaptic loss in early Alzheimer's disease and mild cognitive impairment.* Neurobiol Aging, 2006. **27**(10): p. 1372-84.
26. Brun, A., X. Liu, and C. Erikson, *Synapse loss and gliosis in the molecular layer of the cerebral cortex in Alzheimer's disease and in frontal lobe degeneration.* Neurodegeneration, 1995. **4**(2): p. 171-7.
27. Coben, L.A., W. Danziger, and M. Storandt, *A longitudinal EEG study of mild senile dementia of Alzheimer type: changes at 1 year and at 2.5 years.* Electroencephalogr Clin Neurophysiol, 1985. **61**(2): p. 101-12.
28. van der Hiele, K., et al., *EEG correlates in the spectrum of cognitive decline.* Clin Neurophysiol, 2007. **118**(9): p. 1931-9.
29. Stam, C.J., et al., *Small-world networks and functional connectivity in Alzheimer's disease.* Cereb Cortex, 2007. **17**(1): p. 92-9.
30. Stam, C.J., et al., *Generalized synchronization of MEG recordings in Alzheimer's Disease: evidence for involvement of the gamma band.* J Clin Neurophysiol, 2002. **19**(6): p. 562-74.
31. Harrison, J., et al., *A neuropsychological test battery for use in Alzheimer disease clinical trials.* Arch Neurol., 2007. **64**(9): p. 1323-9.
32. Lannfelt L, B.K., Zetterberg H et al., *Safety, efficacy, and biomarker findings of PBT2 in targeting AB as a modifying therapy for Alzheimer's disease: a phase IIa, double-blind, randomised, placebo-controlled trial.* Lancet, 2008 online. **July 29th**.
33. Rey, A., *L'examen clinique en psychologie.* Paris: Presses Universitaires de France, 1964.
34. Wechsler, D., *Wechsler Memory Scale Manual.* San Diego, 1987. **Psychological Corp**.
35. Reitan, R.M., *The relation of the trail making test to organic brain damage.* J Consult Psychol, 1955. **19**(5): p. 393-4.
36. Reitan, R.M., *Validity of the trail making test as an indicator of organic brain damage.* Perceptual and Motor Skills, 1958. **8**: p. 271-276.
37. Armitage, S.G., *An analysis of cetain psychological tests used for the evaluation of brain injury.* Psychological Monographs, 1946. **60**(1): p. whole no. 277.
38. Lezak, M.D., D.B. Howieson, and D.W. Loring, *Neuropsychological Assessment*. 2004, New York: Oxford University Press.
39. Rosen, W.G., R.C. Mohs, and K.L. Davis, *A new rating scale for Alzheimer's disease.* Am J Psychiatry, 1984. **141**(11): p. 1356-64.
40. van der Elst, W., et al., *The Letter Digit Substitution Test: normative data for 1,858 healthy participants aged 24-81 from the Maastricht Aging Study (MAAS): influence of age, education, and sex.* J Clin Exp Neuropsychol, 2006. **28**(6): p. 998-1009.
41. Laurin, D., et al., *Physical activity and risk of cognitive impairment and dementia in elderly persons.* Arch Neurol, 2001. **58**(3): p. 498-504.
42. Bays, H.E., *Safety considerations with omega-3 fatty acid therapy.* Am J Cardiol, 2007. **99**(6A): p. 35C-43C.
43. Harris, W.S., *Expert opinion: omega-3 fatty acids and bleeding-cause for concern?* Am J Cardiol, 2007. **99**(6A): p. 44C-46C.
44. Scheltens P, K.P., Verhey FRJ, Olde Rikkert MGM, Wurtman RJ, Wilkinson D, Twisk JWR, Kurz A., *Efficacy of a medical food in mild Alzheimer's disease: a randomized, controlled trial.* Alzheimer's & Dementia, 2010. **6**: p. 1-10.
45. Scheltens, P., et al., *Efficacy of a medical food in mild Alzheimer's disease: A randomized, controlled trial.* Alzheimers Dement. **6**(1): p. 1-10 e1.

46. McKhann, G., et al., *Clinical diagnosis of Alzheimer's disease: report of the NINCDS-ADRDA Work Group under the auspices of Department of Health and Human Services Task Force on Alzheimer's Disease.* Neurology, 1984. **34**(7): p. 939-44.

APPENDIX I SCHEDULE OF ASSESSMENTS

n = 40 patients	Screening visit	Baseline visit	6-week call	12-week visit	18-week call	24-week visit	Follow-up call 2 weeks after 24-week visit	End of study visit for patients who discontinued
	Screening within 2 weeks before Baseline or combined							
Informed consent	x							
Eligibility criteria	x	x						
MRI or CT scan	x*							
Medical history/ coexisting disease	x	x						
AE recording			x	x	x	x	x	x
Medication and supplement recording	x	x	x	x	x	x	x	x
Patient characteristics	x							
Vital signs		x				x		x
Blood sample for safety and efficacy		x				x		x
Neuropsychological Test Battery (NTB)		x		x		x		x
EEG		x		x		x		x
MEG		x		x		x		x
Randomisation		x						
Product accountability and diary collection				x		x		x
Product and diary dispensing		x		x				
*In case a MRI or CT scan <2 years from baseline is not available								

APPENDIX II NINCDS-ADRDA CRITERIA

Probable Alzheimer´s disease according to NINCDS-ADRDA criteria [46]

Criteria for the clinical diagnosis of PROBABLE Alzheimer`s disease
- Dementia established by clinical examination and documented by the Mini-Mental Test; Blessed Dementia Scale, or some similar examination, and confirmed by neuropsychological tests;
- Deficits in two or more areas of cognition;
- Progressive worsening of memory and other cognitive functions;
- No disturbance of consciousness;
- Onset between ages 40 and 90, most often after age 65; and
- Absence of systemic disorders or other brain diseases that in and of themselves could account for the progressive deficits in memory and cognition

The diagnosis of PROBABLE Alzheimer`s disease is supported by:
- progressive deterioration of specific cognitive functions such as language (aphasia), motor skills (apraxia), and perceptions (agnosia);
- impaired activities of daily living and altered patterns of behavior;
- family history of similar disorders, particularly if confirmed neuropathologically; and
- laboratory results of:
- normal lumbar puncture as evaluated by standard techniques,
- normal pattern or non-specific changes in EEG, such as increased slow-wave activity, and
- evidence of cerebral atrophy on CT with progression documented by serial observation

Other clinical features consistent with the diagnosis of PROBABLE Alzheimer`s disease, after exclusion of causes of dementia other than Alzheimer`s disease, include:
- plateaus in the course of progression of the illness;
- associated symptoms of depression, insomnia, incontinence, delusions, illusions, hallucinations, catastrophic verbal, emotional, or physical outbursts, sexual disorders, and weight loss;
- other neurologic abnormalities in some patients, especially with more advanced disease and including motor signs such as increased muscle tone, myoclonus, or gait disorder;
- seizures in advanced disease; and
- CT normal for age

Features that make the diagnosis of PROBABLE Alzheimer`s disease uncertain or unlikely include:
- sudden, apoplectic onset;
- focal neurologic findings such as hemiparesis, sensory loss, visual field deficits, and in coordination early in the course of the illness; and
- seizures or gait disturbances at the onset or very early in the course of the illness
